# Effect of *Portulaca oleracea* Addition in Health Care Sand on Apparent Nutrient Digestibility, Serum Parameters, and Excreta Microbiota Metabolism in Tumbler Pigeons

**DOI:** 10.3390/ani15223349

**Published:** 2025-11-20

**Authors:** Hu Li, Jian Zhang, Haiying Li, Xiaobin Li, Ping Zhang, Xinsheng Guo, Jianwei Lin, Kunyu Liao, Lifeng Ke

**Affiliations:** 1 College of Animal Science, Xinjiang Agricultural University, Urumqi 830052, China; lh18690822117@163.com (H.L.); 18723181520@163.com (J.Z.); lhy-3@163.com (H.L.); pingzhangxau@163.com (P.Z.); 13017602713@163.com (X.G.); 18324052590@163.com (J.L.); 18915391126@163.com (K.L.); 2 Yanqi County Lifeng Rare Poultry Breeding Professional Cooperative, Korla 841100, China; 17839750693@163.com

**Keywords:** *Portulaca oleracea*, tumbler pigeons, apparent nutrient digestibility, antioxidant capacity, microbiota metabolism

## Abstract

Tumbling pigeons are prone to oxidative stress and disruption of gut microbiota balance during long-term exercise training and competitions. Considering that *Portulaca oleracea* (*P. oleracea*), as a natural plant feed additive, has natural antioxidant, anti-inflammatory, and gut function improvement effects. This study investigates the effects of adding *P. oleracea* into health care sand on nutrient digestion and metabolism, serum parameters, and excreta microbiota metabolism in tumbler pigeons. Ninety 12-month-old tumbler pigeons were randomly assigned to three groups, with ten cages with three birds each. The CON Group received a basal diet added with 4 g of health care sand; Group TRT1 received a basal diet added with 4 g of health care sand containing 0.75% *P. oleracea*; and Group TRT2 received a basal diet added with 4 g of health care sand containing 1.00% *Portulaca oleracea*. The adaptation period lasted for 7 days, followed by a formal testing phase of 45 days. All tumbler pigeons received 1 h of flight training daily. The CON and TRT2 groups showed significantly increased dry matter (DM) apparent digestibility by 11.68% (*p* < 0.01) and 8.50% (*p* < 0.05), respectively, compared to the TRT1 group. The TRT2 group also demonstrated higher organic matter (OM) apparent digestibility (increase of 4.25%, *p* < 0.05) and markedly improved crude protein (CP) digestibility (16.72% higher than CON, *p* < 0.05; 27.12% higher than TRT1, *p* < 0.01). Both gross energy (GE) and metabolizable energy (ME) digestibility were significantly elevated in CON and TRT2 groups compared to TRT1 (*p* < 0.01). Compared to the CON group, the TRT2 group showed a 19.86% decrease in lactate (LAC) level (*p* < 0.05) alongside a 38.91% increase in lactate dehydrogenase (LDH) activity (*p* < 0.05). Serum uric acid (UA) levels increased by 33.65–36.14% in both treatment groups (*p* < 0.05). Antioxidant capacity markedly improved, with malondialdehyde (MDA) decreasing by up to 27.75% (*p* < 0.01) and key antioxidant enzymes superoxide dismutase (SOD), glutathione peroxidase (GSH-Px), catalase (CAT), and total antioxidant capacity (T-AOC) showing dose-dependent enhancements of up to 25.23% (*p* < 0.01). Other serum biochemical parameters showed no significant differences (*p* > 0.05). Microbial analysis demonstrated that Actinobacteriota, Acidothermaceae, and Nitrosotaleaceae were enriched in the TRT1 and TRT2 groups, while the relative abundance of Proteobacteria and Chitinophagaceae decreased (*p* > 0.05). Metabolomic analysis revealed a significant increase in beneficial metabolites, including agmatine, pyropheophorbide-a, and *N*-acetylmuramate (*p* < 0.01). In conclusion, the addition of 1.00% *Portulaca oleracea* in health care sand effectively enhanced apparent nutrient digestibility, improved antioxidant capacity, and modulated the intestinal microbiota and metabolic profile of tumbler.

## Simple Summary

This study demonstrates that adding health care sand with 1% *Portulaca oleracea* effectively enhances the metabolic performance of tumbler pigeons. The results show that this natural additive significantly improves nutrient utilization efficiency, boosts antioxidant capacity, and optimizes gut microbiota structure—particularly by promoting beneficial bacteria such as Actinobacteria. These comprehensive improvements work together to provide better metabolic support and anti-fatigue capacity during high-intensity flight training, offering a reliable nutritional foundation for enhanced athletic performance in tumbler pigeons.

## 1. Introduction

The tumble pigeon originated from the *Columba livia* and is characterized by its unique genetic trait of performing somersaults during flight [1]. The Classic of Pigeons, document the distinctive aerial behavior of tumbler pigeons, which fly in dense flocks and continuously roll in midair [2]. In China, tumbler pigeons are regarded as an important ornamental breed, admired for their remarkable flight speed and frequent aerial tumbling performance. The “loft competition” (public loft race) represents their primary form of competitive sport. Sustained high-altitude flight and stable tumbling ability are critical determinants of performance success in tumbler pigeons. In addition to genetic factors, management practices, flight training intensity, and nutritional regulation play crucial roles in shaping flight performance. Flight training places high energy and metabolic demands on pigeons, requiring a constant nutrient supply for both aerobic and anaerobic metabolism. However, prolonged or intensive training can cause oxidative stress, leading to excess reactive oxygen species, lactate buildup, lipid peroxidation, and weakened antioxidant defenses. These effects result in fatigue, inflammation, and gut microbiota imbalance, ultimately impairing health and performance [3,4,5]. Therefore, enhancing antioxidant stress resistance and maintaining the dynamic balance of gut microbiota are crucial for improving athletic training levels and individual health, all while ensuring a foundation of scientific exercise training.

With the growing emphasis on green and sustainable animal farming, natural plant-derived feed additives from traditional Chinese medicine (TCM) have gained increasing attention. These additives combine nutritional and pharmacological benefits, offering a safe, residue-free, and eco-friendly alternative for modern animal production. Consequently, the use of Chinese herbal medicines as functional feed additives has become a major focus in animal nutrition research [6,7].

*Portulaca oleracea* (*P. oleracea*), is an annual succulent herb belonging to the family *Portulacaceae*. It is widely distributed across temperate and tropical regions of the world and is also referred to as “horse tooth herb” or “longevity vegetable” in China, owing to its leaf shape resembling horse teeth and its slippery texture similar to that of *Amaranthus* [8]. *P. oleracea* is a traditional Chinese plant recognized for its edible and medicinal properties. It serves as a remedy in traditional medicine to alleviate symptoms associated with various ailments [9]. According to traditional Chinese medicine, *P. oleracea* is considered non-toxic and possesses several functions, including clearing heat, reducing swelling, detoxifying, halting bleeding, eliminating dampness, treating dysentery, and combating parasites. No significant side effects have been reported [10]. Modern phytochemical and pharmacological studies have revealed that *P. oleracea* contains a variety of bioactive compounds, including flavonoids, polysaccharides, and alkaloids, which confer multiple biological activities such as antibacterial, antioxidant, anti-inflammatory, hypoglycemic, and antitumor effects [11,12]. Alkaloids and flavonoids contribute predominantly to its antimicrobial and anti-inflammatory actions [13,14], while soluble non-starch polysaccharides demonstrate potent antioxidant and immunomodulatory activities [15,16]. Moreover, the soluble dietary fibers of *P. oleracea* promote the proliferation of beneficial intestinal microbiota, help maintain gut microbial homeostasis, enhance nutrient digestion and absorption, and inhibit the growth of pathogenic bacteria [17]. Owing to these properties, *P. oleracea* exhibits antimicrobial potential and may complement strategies aimed at minimizing antibiotic use in animal production. However, no studies have examined the effect of *P. oleracea* on nutrient utilization, antioxidant status, and intestinal microbiota in performance pigeons. Therefore, this study investigated the effects of dietary *P. oleracea* addition via health care sand on nutrient digestibility and metabolism, serum biochemical and antioxidant parameters, and excreta microbial composition in tumbling pigeons. The findings aim to provide theoretical and practical insights into improving the anti-stress performance of racing pigeons during training through natural herbal addition.

## 2. Materials and Methods

### 2.1. Animal Ethics Statement

This study was reviewed and granted by the Institutional Animal Care and Use Ethics Committee of Xinjiang Agricultural University (Urumqi, China; protocol permit number: 2020024).

### 2.2. Experimental Animals and Design

Twelve-month-old pigeons were selected for this study as they represent young adults that have completed their growth yet are at a prime age for intensive flight training. This ensures that the findings are directly relevant to the physiological state of pigeons typically used in competitive tumbling. A total of 90 healthy 12-month-old tumbling pigeons were used in this study. A stratified randomization procedure was applied to assign the pigeons to one of three treatment groups, using body weight and baseline flight performance as stratification factors to ensure baseline equivalence. The random allocation sequence was generated using Microsoft Excel. Following group assignment, the pigeons were housed in replicate subgroups, with each treatment group comprising 10 cages of three birds each. (1) CON group: basal diet +4 g health care sand; (2) TRT1 group: basal diet +4 g health care sand containing 0.75% *P. oleracea* powder; (3) TRT2 group: basal diet +4 g health care sand containing 1.00% *P. oleracea* powder. After an adaptation period of 7 days, the trial lasted for 45 days. From the beginning of the adaptation period until the end of the trial, all tumbling pigeons underwent one hour of flight training per day. All experimental birds were obtained from Yanqi County Lifeng Rare Poultry Breeding Professional Cooperative (Korla, China) The experimental pigeons underwent daily flight training at 17:00, conducted in groups within a circular open-field testing area with a diameter of 200 m for free flight. The experimenters simultaneously applied two external signals: a visual signal (fag-waving) and an auditory signal (whistle sound). The stimulation protocol was continuously applied from the start of release until the test group had flown for 1 h. The flight duration was defined as the interval from the moment of take-off to the moment of landing. To quantify flight ability, we primarily assessed the total flight duration (from take-off to landing) during each daily session. Before the formal experiment, we conducted a baseline assessment to ensure homogeneity across groups. The total flight duration of each pigeon from this baseline test was analyzed using one-way analysis of variance (ANOVA). This statistical comparison confirmed that there were no significant differences in initial flight ability among the three treatment groups. The temperature in the dovecote was kept at around 25 °C. In mid-June, the plant material of wild *P. oleracea* was collected from the cropland of the Yanqi (Xinjiang, China). Firstly, fresh *P. oleracea* leaves were picked, washed, and dried in the shade. Dried *P. oleracea* leaves were then crushed and sifted for tumbling pigeons’ feed.

The nutritional composition was determined according to the official Chinese national standards for *P. oleracea* analysis. Crude protein (CP) was determined by the Kjeldahl method (GB/T 6432-1994) [18]. Crude fat (EE) was analyzed by the Soxhlet extraction method (GB/T 6433-2006) [19]. Neutral detergent fiber (NDF) and acid detergent fiber (ADF) were determined using the methods described by Van Soest et al. [20]. Determine the calcium and phosphorus content using the method in (GB/T 6437-2002) [21]. The powder (60 g) was extracted with 300 mL of aqueous ethanol (80% *v*/*v*) in a Soxhlet apparatus for 72 h. After extraction, the solvent was filtered and evaporated using a Rotavapor. The purslane ethanolic extract so obtained was stored at −20 °C until further use. Total polyphenols were measured by Folin–Ciocalteu method according to a reference [22]. The content of soluble non-starch polysaccharide was analyzed by phenol–sulfuric acid method according to a reference [23]. The total flavonoid content was assessed using the aluminum chloride colorimetric method according to a reference [24]. *P. oleracea* powder contained 16.89% crude protein, 2.14% crude fat, 41.24% NDF, 30.85% ADF, 1.78% calcium, and 0.57% phosphorus on dry matter basis. The contents of total phenols, soluble non-starch polysaccharides and total flavonoids as the main compounds in the ethanolic extract of dry *P. oleracea* were 13.78 mg/g, 51.61 mg/g and 4.87 mg/g, respectively. Table 1 shows the ingredients and nutritional content of the basal diet and Table 2 shows the composition of health care sand.

### 2.3. Sample Collection

On the final day of the experiment, blood samples were collected using non-heparinized syringes and placed in sterile vials. The samples were centrifuged at 3000 rpm for 10 min to separate serum, which was then stored at −20 °C for further biochemical analysis. Serum samples from three pigeons within each replicate were pooled into one composite sample, resulting in 10 samples per group. On the same day, fresh excreta samples were collected from each group under hygienic conditions in the morning. The samples were immediately frozen in liquid nitrogen and then stored at −80 °C until further analysis of gut microbiota composition and metabolite profiles.

### 2.4. Sampling and Determination of Nutrient Digestibility and Metabolism

The total excreta collection method was used to evaluate nutrient digestibility and metabolism in pigeons. During the final week of the experiment, two healthy pigeons were randomly selected from each replicate and individually housed in metabolism cages for digestibility trials. Excreta samples from two birds were pooled to form one composite sample per replicate, resulting in a total of ten samples per treatment group for subsequent analyses. Before the formal sampling, pigeons were fasted for 24 h to clear the intestinal contents. Thereafter, each bird was fed 20 g of the experimental diet per day for three consecutive days. All excreta excreted over the 3-day collection period were collected, pooled per cage, and used as representative samples. Feed and air-dried excreta samples were oven-dried at 65 °C to a constant weight, ground, and passed through a fine mesh for chemical analysis. Excreta samples were air-dried prior to oven-drying to prevent crust formation, minimize the loss of volatile compounds, and inhibit microbial activity, thereby ensuring the accuracy of subsequent chemical analyses.

The contents of dry matter (DM), organic matter (OM), metabolizable energy (ME), crude protein (CP), and ether extract (EE) were determined in excreta samples, while DM, OM, ME, CP, EE, and crude fiber (CF) were analyzed in feed samples. Analytical methods followed the Chinese national standards: DM: GB/T 6435-2014 [25]; OM: GB/T 6438-2007 [26]; GE: GB/T 14489.1-2008 [27]; CP: GB/T 6432-1994 [18]; EE: GB/T 6433-2006 [19]. The formula for calculating metabolic energy is ME (kcal/kg DM) = [GE intake − (GE excreta + GE urine)]/Feed DM intake.

### 2.5. Serum Parameters

Serum biochemical indices were determined using commercial colorimetric assay kits. The analyzed parameters included total protein (TP), albumin (ALB), globulin (GLB), aspartate aminotransferase (AST), alanine aminotransferase (ALT), alkaline phosphatase (ALP), uric acid (UA), total cholesterol (TC), triglycerides (TG), glucose (GLU), lactate (LAC), and lactate dehydrogenase (LDH). Antioxidant-related parameters in serum included malondialdehyde (MDA), superoxide dismutase (SOD), glutathione peroxidase (GSH-Px), total antioxidant capacity (T-AOC), and catalase (CAT). All assays were performed following the manufacturer’s instructions using commercial kits purchased from Nanjing Jiancheng Bioengineering Institute (Jiangsu, China).

### 2.6. Excreta Microbial Community

A Stool DNA Kit (Omega Bio-tek, Norcross, GA, USA) was used to extract whole genomic DNA from excreta samples of tumbler pigeons. The V3 to V4 region of the 16S rRNA gene was amplified using the primers 338F (5′-ACTCCTACGGGAGGCAGCA-3′) and 806R (5′-GGACTACHVGGGTWTCTAAT-3′) [28]. The samples were separated on a 2% agarose gel electrophoresis and recovered with the AxyPrep DNA Gel Extraction Kit (Axygen Biosciences, Union City, CA, USA). On the Illumina MiSeq platform, the purified amplicons were pooled and paired-end sequenced. UPARSE software (v11.0.667) was used to cluster operational taxonomic units (OTU) based on 97% sequence similarity. The RDP Classifier determined the taxonomy of each OTU representative sequence with a confidence level greater than 70%. Taxonomic annotation and relative abundance of microbial communities at the phylum and genus levels were visualized as bar plots using R software (v3.6.0). Differences in excreta microbiota composition among groups were identified using linear discriminant analysis effect size (LEfSe), with a linear discriminant analysis (LDA) score threshold of >2. Predicted functional profiles of the excreta microbiota were inferred using PICRUSt2 (v2.6.2) to explore potential differences in microbial metabolic pathways among treatments.

### 2.7. Metabolites Extraction, UHPLC-MS/MS, and Metabolomic Analysis

The extraction of metabolites from excreta samples was performed following a previously described method with minor modifications [29]. Briefly, the excreta samples (100 mg) were individually ground with liquid nitrogen, and the homogenate was resuspended in pre-chilled 80% methanol using vortex mixing. The samples were incubated on ice for 5 min, followed by centrifugation at 15,000× *g* at 4 °C for 20 min. A portion of the supernatant was diluted with LC-MS-grade water to achieve a final concentration of 53% methanol. The samples were then transferred to fresh Eppendorf tubes and centrifuged again at 15,000× *g* at 4 °C for 20 min. Finally, the supernatant was injected into the LC-MS/MS system for analysis.

UHPLC-MS/MS analyses were carried out based on established protocols [30]. The instrumental analyses were performed using a Vanquish UHPLC system (Thermo Fisher, Bremen, Germany) coupled with an Orbitrap Q ExactiveTM HF mass spectrometer or Orbitrap Q ExactiveTM HF-X mass spectrometer (Thermo Fisher, Germany) at Novogene Co., Ltd. (Beijing, China). The samples were injected onto a Hypersil Gold column (100 × 2.1 mm, 1.9 µm) and analyzed using a 12 min linear gradient at a flow rate of 0.2 mL/min. The eluents for both the positive and negative polarity modes were eluent A (0.1% FA in water) and eluent B (methanol). The solvent gradient was as follows: 2% B for 1.5 min; 2–85% B for 3 min; 85–100% B for 10 min; A linear gradient from 100% B to 2% B was applied over 10.1 min; and 2% B at 12 min. The Q ExactiveTM HF mass spectrometer was operated in positive/negative polarity mode with a spray voltage of 3.5 kV, a capillary temperature of 320 °C, a sheath gas flow rate of 35 psi, an aux gas flow rate of 10 L/min, and an S-lens RF level of 60, with an aux gas heater temperature of 350 °C.

The raw data files generated by UHPLC-MS/MS were processed using Compound Discoverer 3.3 (CD3.3, Thermo Fisher) for peak alignment, peak picking, and the quantification of each metabolite. The key parameters were set as follows: peak area correction was performed with the first QC, mass tolerance was set to 5 ppm, signal intensity tolerance was set to 30%, and a minimum intensity threshold was applied. Subsequently, the peak intensities were normalized to the total spectral intensity. The normalized data were used to predict molecular formulas based on additive ions, molecular ion peaks, and fragment ions. The peaks were then matched against the mzCloud (https://www.mzcloud.org/ accessed on 19 March 2025), mzVault, and MassList databases to obtain accurate qualitative and relative quantitative results. Statistical analyses were conducted using R (version R-3.4.3), Python (version 2.7.6), and CentOS (release 6.6). When the data were not normally distributed, they were standardized using the following formula: sample raw quantitation value/(sum of sample metabolite quantitation values/sum of QC1 sample metabolite quantitation values). This yielded the relative peak areas. Compounds with a CV of relative peak areas greater than 30% in the QC samples were excluded, and the final metabolites’ identification and relative quantification results were obtained.

### 2.8. Statistical Analysis

All data were analyzed using IBM SPSS Statistics 26.0 software (SPSS Inc., Chicago, IL, USA). In this study, all data were tested and presented as a normal distribution. Differences among the control and treatment groups were evaluated by one-way analysis of variance (ANOVA), followed by Duncan’s multiple range test for post hoc comparisons. The linear mixed-effects model was constructed as follows: *Y_ij_* = *µ* + *D_i_* + *P_j_* + *e_ij_*, where *μ* represents the overall mean, *D_i_* is the fixed effect of diet (*i* = control, TRT1, TRT2), *P_j_* is the random effect of individual animals to account for variation between pigeons within the same cage, and *e_ij_* denotes the residual error. Differences in the relative abundance of excreta bacteria were analyzed using the Wilcoxon rank-sum test. Functional differences in predicted microbial metabolism were determined using Welch’s *t*-test in STAMP software (v2.1.3)**.** For apparent nutrient digestibility analysis, the sample size was *n* = 10 (one composite sample per cage). For excreta microbiota and metabolomic analyses, one composite excreta sample per cage (*n* = 10 biological replicates per group) was subjected to 16S rRNA sequencing and LC-MS/MS, respectively. Statistical significance was declared at *p* < 0.05, while 0.05 < *p* < 0.10 was considered a tendency toward significance.

## 3. Results

### 3.1. Effects of P. oleracea Addition in Health Care Sand on Apparent Nutrient Digestibility and Metabolism in Tumbler Pigeons

The effects of *P. oleracea* addition in health care sand on the apparent nutrient digestibility and metabolism of tumbler pigeons are presented in Table 3 and Table S1. As shown in Table 3, compared with the TRT1 group, the apparent digestibility of DM in the CON and TRT2 groups increased by 11.68% (*p* < 0.01) and 8.50% (*p* < 0.05), respectively; compared with TRT1, the OM digestibility in the TRT2 group increased by 4.25% (*p* < 0.05); for CP apparent digestibility, the TRT2 group was higher than the CON group by 16.72% (*p* < 0.05) and higher than the TRT1 group by 27.12% (*p* < 0.01). The digestibility of GE and ME in the CON and TRT2 groups was higher than in the TRT1 group by 3.58%, 3.92% and 3.62%, 3.91%, respectively (*p* < 0.01). There were no significant differences in EE digestibility among the groups (*p* > 0.05).

### 3.2. Effects of P. oleracea Addition in Health Care Sand on Serum Biochemical Parameters and Antioxidant Capacity in Tumbler Pigeons

**Figure 1:**
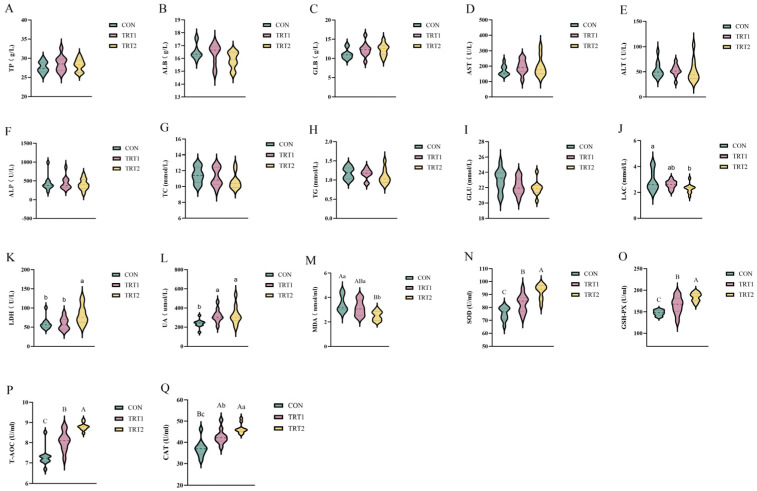
Effects of *P. oleracea* addition in health care sand on serum biochemical and antioxidant capacity in tumbler pigeons. (**A**) TP, total protein; (**B**) ALB, albumin; (**C**) GLB, globulin; (**D**) Aspartate aminotransferase, AST; (**E**) Alanine aminotransferase, ALT; (**F**) Alkaline phosphatase, ALP; (**G**) Total cholesterol, TC; (**H**) Triglycerides, TG; (**I**) Glucose, GLU; (**J**) Lactate, LAC; (**K**) Lactate dehydrogenase, LDH; (**L**) Uric acid, UA; (**M**) Malondialdehyde, MDA; (**N**) Superoxide dismutase, SOD; (**O**) Glutathione peroxidase, GSH-Px; (**P**) Total antioxidant capacity, T-AOC; (**Q**) Catalase, CAT. Values with different lowercase letters indicate significant differences (^a, b, c^: *p* < 0.05), and those with different uppercase letters indicate highly significant differences (^A, B, C^: *p* < 0.01). Data are presented as mean ± standard deviation (*n* = 10).

The effects of *P. oleracea* addition in health care sand on serum biochemical and antioxidant capacity in tumbler pigeons are shown in Figure 1 and Table S2. Compared with the CON group, the LAC level in the TRT2 group decreased by 19.86 (*p* < 0.05); serum LDH activity in the TRT2 group was 38.91% and 39.67% higher than in the CON and TRT1 groups, respectively (*p* < 0.05); serum UA levels in TRT1 and TRT2 were 33.65% and 36.14% higher than the CON group (*p* < 0.05); compared with the CON group, MDA levels in the TRT1 and TRT2 groups decreased by 9.25% (*p* < 0.05) and 27.75% (*p* < 0.01), respectively; compared with the CON group, the activities of SOD, GSH-Px, and T-AOC in the TRT1 and TRT2 groups increased by 10.39% and 22.76%, 12.04% and 24.96%, 10.97% and 20.71%, respectively (*p* < 0.01); compared with the CON group, CAT activity in the TRT1 and TRT2 groups increased by 15.20% and 25.23% (*p* < 0.01), and the TRT2 group was 8.70% higher than TRT1 (*p* < 0.05). No significant differences were observed in other serum biochemical indicators (*p* > 0.05).

### 3.3. Microbial Composition and Function of Excreta

**Figure 2:**
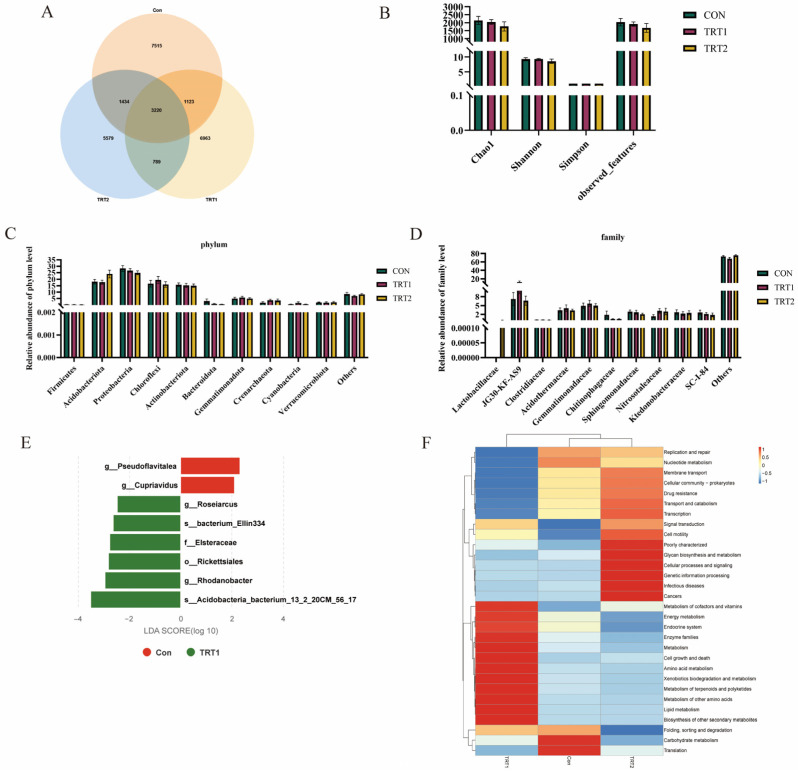
Effects of *P. oleracea* addition in health care sand on the microbial community composition in excreta of tumbler. (**A**) OTU Venn diagram. (**B**) α-diversity indices (Chao1, Shannon, Simpson). (**C**) Phylum-level composition. (**D**) Family-level composition. (**E**) LEfSe analysis identifying differential bacteria (LDA > 2, *p* < 0.05). (**F**) PICRUSt2-predicted functional profiles (red/blue indicate higher/lower enrichment) (*n* = 10).

A total of 26,650 OTUs were detected across all excreta samples. Among these, 3217 OTUs were shared among the three groups, 7541 OTUs were unique to the control group, and 6962 and 5575 OTUs were specific to TRT1 and TRT2, respectively (Figure 2A). Based on α-diversity analysis, no statistically significant differences (*p* > 0.05) were observed in Chao1, Shannon, or Simpson indices among groups (Figure 2B).

Phylum-level analysis revealed Proteobacteria, Actinobacteriota, Chloroflexi, and Acidobacteria as the dominant bacterial groups (Table S3). The addition of *P. oleracea* feeding showed a trend toward reducing Proteobacteria abundance in tumbler excreta. Compared to the CON and TRT1 groups, the TRT2 group exhibited a trend toward increased Actinobacteriota abundance (*p* > 0.05; Figure 2C). At the family level (Table S4), JG30KFAS9, Acidothermaceae, and Gemmatimonadaceae were the dominant bacterial groups at the family level. Compared with the CON and TRT2 groups, the abundance of Acidothermaceae in tumbler excreta from the TRT1 group showed an increasing trend. The abundance of Chitinophagaceae in the CON group was higher than that in the purslane-added groups, while the relative abundance of Nitrosotaleaceae showed a decreasing trend (*p* > 0.05; Figure 2D).

To further identify differences in excreta microbial communities between groups, LEfSe (Linear discriminant analysis effect size) analysis was employed to identify species exhibiting statistically significant differences between groups at various taxonomic levels. As shown in Figure 2E, eight significantly different species were identified across various taxonomic levels between the CON and TRT1 groups. Among these, the CON group exhibited six significantly different species: g_Pseudoflavitalea and g_Cupriavidus. The TRT1 group showed six significantly different species: *s_Acidobacteria_bacterium_13_2_20CM_56_17*, g_Rhodanobacter, o_Rickettsiales, f_Elsteraceae, *s_bacterium_Ellin334*, and g_Roseiarcus. Further analysis of PICRUSt2 functional prediction for excreta microbiota revealed that the TRT1 group showed positive correlations with four functions: Ribosome biogenesis, Pyruvate metabolism, Carbon fixation pathways in prokaryotes, and Quorum sensing. The CON group showed positive correlations with six functions: Oxidative phosphorylation, Exosome, Protein kinases, Peptidases, Glyoxylate and dicarboxylate metabolism, Lipid biosynthesis proteins, and Glycine, serine and threonine metabolism (Figure 2F).

### 3.4. Metabolomics Analysis Results

#### 3.4.1. OPLS-DA Analysis

**Figure 3:**
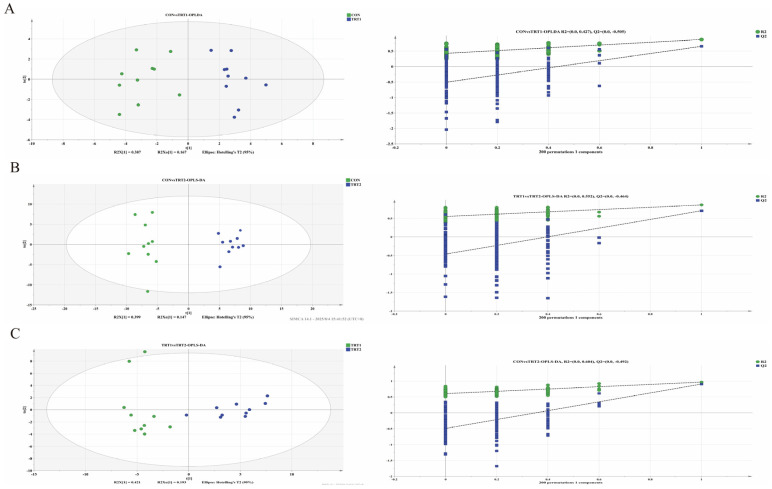
OPLS-DA score plots (**left**) and corresponding validation plots (**right**) for pairwise comparisons. (**A**–**C**) Between the CON vs. TRT1, CON vs. TRT2, and TRT1 vs. TRT2 comparisons, respectively. Abbreviation: OPLS-DA, Orthogonal Projection of Latent Structures Discriminant Analysis (*n* = 10).

Non-targeted metabolomics analysis was employed to evaluate metabolic differences in the gut microbiota. Pairwise comparisons were conducted between the CON group, TRT1, and TRT2, validated using an orthogonal partial least squares discriminant analysis (OPLS-DA) model (Figure 3A–C). The R^2^Y (cum) and Q^2^ (cum) values for OPLS-DA across all groups exceeded 0.4, indicating stable and accurate predictions from the model. In the OPLS-DA scoreplot, excreta samples from each group were completely separated. Furthermore, in the randomization test, Q^2^ values on the Y-axis were consistently negative, indicating the model’s strong predictive capability and absence of overfitting. This material demonstrated sufficient reproducibility, making it suitable for subsequent qualitative and quantitative validation analyses.

#### 3.4.2. Metabolite Classification and Differential Metabolite Analysis

**Figure 4:**
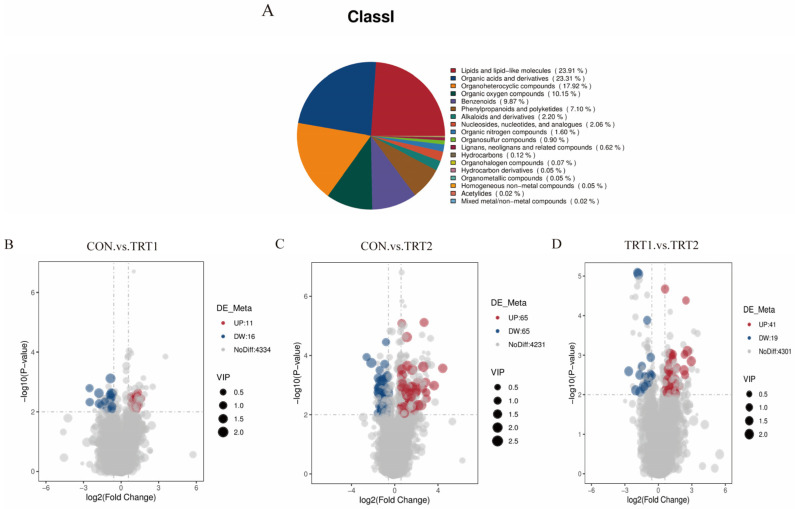
Excreta metabolomic analysis showing: (**A**) Overall metabolite classification. (**B**–**D**) Volcano plots of differentially expressed metabolites between the CON vs. TRT1, CON vs. TRT2, and TRT1 vs. TRT2 groups, respectively (*n* = 10).

A total of 4361 metabolites with known structures were identified in this study, categorized into 18 classes. Lipids and lipid-like molecules accounted for 23.91%, organic acids and derivatives for 23.31%, organoheterocyclic compounds for 17.92%, organic oxygen compounds for 10.15%, benzenoids (9.87%), phenylpropanoids and polyketides (7.10%), with the remaining 7.74% comprising other categories (Figure 4A). Differential metabolites were further identified based on OPLS-DA criteria, including fold change (FC) ≥ 1.5 or FC ≤ −0.67, *p* < 0.05, and VIP > 1. The screening results were visually represented using a volcano plot. The results revealed 27 differentially expressed metabolites (11 up-regulated, 16 down-regulated) in the CON vs. TRT1 group (Figure 4B), 130 metabolites (65 up-regulated, 65 down-regulated) in the CON vs. TRT2 group (Figure 4C), and 60 differentially expressed metabolites (41 up-regulated and 19 down-regulated) were identified in the TRT1 vs. TRT2 group (Figure 4D).

#### 3.4.3. KEGG Classification and Enrichment Pathway Analysis of Differentially Expressed Metabolites

**Figure 5:**
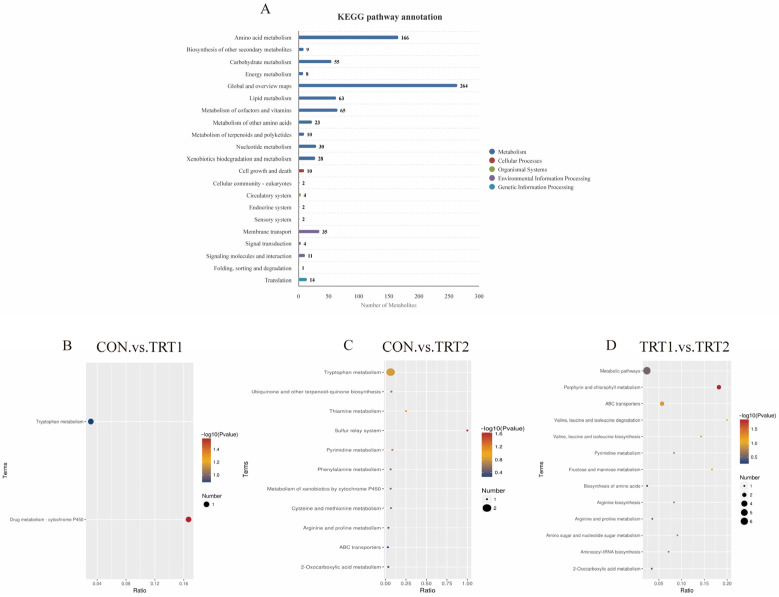
KEGG pathway enrichment analysis of differentially expressed metabolites. (**A**) KEGG classification of differentially expressed metabolites. (**B**–**D**) Bubble plots show significantly enriched pathways for the CON vs. TRT1, CON vs. TRT2, and TRT1 vs. TRT2 comparisons, respectively. Spot color and size denote the *p*-value significance and the number of metabolites involved (*n* = 10).

Comparison of differentially expressed metabolites with the KEGG database revealed that their biological functions primarily relate to Globat and overview maps, Amino acid metabolism, Metabolism of cofactors and vitamins, and Lipid metabolism (Figure 5A). Enrichment analysis of metabolic pathways revealed that: The CON group and the TRT1 group showed significant differences in Tryptophan metabolism and Drug metabolism–cytochrome P450 (Figure 5B); The CON group and TRT2 group exhibited differences in metabolites primarily enriched in pathways such as Tryptophan metabolism (Figure 4C); In the TRT1 and TRT2 groups, metabolic pathways such as Porphyrin and chlorophyll metabolism and ABC transporters were significantly enriched (Figure 4D).

#### 3.4.4. Analysis of Highly Significant Differential Metabolites Enriched in the KEGG Pathway

**Figure 6:**
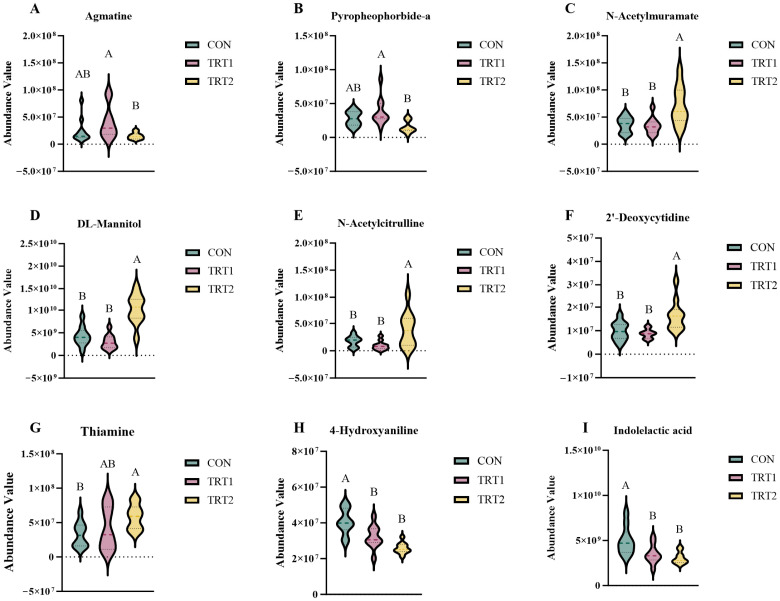
Results of highly significant differential metabolites. (**A**) Agmatine; (**B**) Pyropheophorbide-a; (**C**) *N*-Acetylmuramate; (**D**) DL-Mannitol; (**E**) *N*-Acetyleitrulline; (**F**) 2′-Deoxyeytidine; (**G**) Thiamine; (**H**) 4-Hydroxyaniline; (**I**) Indolelactie acid. Different capital letters of undefined indicate highly significant differences (^A, B^: *p* < 0.01) (*n* = 10).

The metabolites with highly significant differences in the KEGG pathway are shown in Figure 6 and Table S5. The results showed that the levels of Agmatine and Pyropheophorbide-a in the TRT1 group were significantly higher than those in the TRT2 group (*p* < 0.01; Figure 6A,B). However, *N*-Acetylmuramate, DL-Mannitol, and *N*-Acetyleitrulline were lower than those in the TRT2 group (*p* < 0.01; Figure 6C–E); 2′-Deoxyeytidine and Thiamine in the TRT2 group were highly significantly increased (*p* < 0.01; Figure 6F,G); Compared with the CON group, the levels of Indolelactie acid and 4-Hydroxyaniline in the TRT1 and TRT2 groups decreased (*p* < 0.01; Figure 6H,I).

## 4. Discussion

### 4.1. Effects of P. oleracea Addition in Health Care Sand on Apparent Nutrient Digestibility and Metabolism in Tumbler

The apparent digestible metabolic rate of nutrients in poultry diets serves as a comprehensive assessment of both the diet’s composition and the animals’ feed digestion characteristics. In this study, the apparent digestibility rates of OM and CP in the diet of tumbler pigeons in the TRT2 group exhibited an increasing trend compared to the CON group. A study by Abd El-Hack et al. [31], investigated the effects of adding *P. oleracea* extract to a corn-soybean meal-based diet for Japanese quails. The results indicated that the addition of 2 mL/kg of *P. oleracea* significantly enhanced the digestibility coefficient of ether extract (EE) in the quail diet. Furthermore, the digestibility coefficients of CF, DM, and OM at 1, 3, and 4 mL/kg of *P. oleracea* exhibited the highest significance, respectively. Concurrently, the activities of amylase and lipase also increased. Wang et al. [32] reported that the inclusion of *P. oleracea* L. in feed enhances the relative abundance of Lactobacillus in the intestinal tract, regulates the intestinal flora environment, and promotes carbohydrate metabolism. This, in turn, improves growth performance and reduces the feed-to-weight ratio. This study is similar to the results of the aforementioned research. It is also reported that greater digestive enzyme production and/or activity by adding *P. oleracea* can result in an improvement of digestibility and availability of nutrients from feedstuffs [33]. Therefore, the improvement in apparent digestibility of tumbler pigeons fed with added purslane may be partly attributed to changes in digestive enzyme secretion or enhanced digestive capacity. On the other hand, studies have found that the large intestine, as the main site for the fermentation of its soluble polysaccharides, can regulate the gut microbiota and increase the accumulation of short-chain fatty acids. Among them, short-chain fatty acids can provide a primary energy source for intestinal epithelial cells, thereby enhancing the digestion and absorption of nutrients in the gut [34,35]. Notably, the apparent digestibility of DM, OM, CP, and GE levels in the TRT1 group was significantly or highly lower than those observed in both the CON group and the TRT2 group. The reason for this outcome may be related to the dose-effect relationship of *P. oleracea*. After entering the gastrointestinal tract with the feed, *P. oleracea* is preferentially utilized by the microbial community present, leading to partial consumption of its nutritional components and preventing them from fully exerting their biological functions. Subsequently, these components undergo host metabolic processes. These findings further indicate that *P. oleracea*, within an appropriate dosage range, can positively enhance nutrient digestion and absorption in racing pigeons.

### 4.2. Effects of P. oleracea Addition in Health Care Sand on Serum Biochemical Parameters and Antioxidant Capacity in Tumbler Pigeons

The improvement in nutrient digestibility may be related to the regulatory effects of *P. oleracea* on the serum biochemistry and antioxidant capacity of tumbler pigeons. Therefore, we further evaluated the serum biochemical indicators and overall antioxidant capacity of these pigeons. Serum biochemistry serves as a critical indicator for assessing the physiological health status of livestock and poultry. In contrast to studies reporting improved lipid profiles in broilers [36] or hepatoprotective effects in rats [37], our study did not observe significant alterations in routine serum biochemical parameters in tumbler pigeons. This discrepancy could be attributed to interspecies differences, the distinct physiological status of tumbler pigeons, or the specific dosage and composition of the *P. oleracea* used. Consequently, the enhanced nutrient digestion observed in this study is more directly supported by the profound improvements in systemic antioxidant capacity and gut health, rather than by changes in conventional serum biochemistry.

The significantly reduced serum lactate (LAC) and increased lactate dehydrogenase (LDH) activity in *P. oleracea*-added tumbler pigeons indicate an enhanced clearance of exercise-induced lactate. This finding suggests that *P. oleracea* may facilitate the LDH-catalyzed conversion of lactate to pyruvate, promoting its entry into the tricarboxylic acid cycle for energy production [38,39], thereby potentially accelerating recovery from exercise fatigue. Concurrently, the elevated serum uric acid (UA) levels in treatment groups may reflect intensified purine metabolism. We hypothesize that this could be linked to the polyphenols in *P. oleracea* promoting protein turnover and repair processes in muscle tissues following high-intensity flight [40].

Prolonged or high-intensity vigorous exercise can lead to the excessive generation of free radicals. When this exceeds the body’s clearance capacity, it disrupts cellular oxidant-antioxidant balance, causing oxidative stress. This state promotes lipid peroxidation, impairs aerobic metabolism, and damages tissues [41], ultimately contributing to exercise-induced fatigue and compromised athletic performance. The results of this study show that *P. oleracea* significantly increases the activities of SOD, CAT, GSH-Px, and T-AOC in the serum of tumbling pigeons, while reducing MDA activity. Xu et al. [42] found that by adding *P. oleracea* L. additives to the diet of weaned piglets, *P. oleracea* L. could significantly reduce the IL-6 in the blood of weaned piglets. On the 14th day, the activity of serum SOD significantly increased, effectively improving stress-induced growth arrest in piglets. Dahran et al. [43] indicate that dietary *P. oleracea* leaf powder can mitigate the negative effects of lipid peroxidation in the intestinal tissues of Nile tilapia under conditions of prolonged cadmium (Cd) exposure, by enhancing the activities of SOD, CAT, and GSH. This study is consistent with the results mentioned above. Meanwhile, research reports indicate that *P. oleracea* is also regarded as an excellent source of glutathione, which plays a significant role as a substrate for GSH-Px in animal cells. The glutathione present in *P. oleracea* can be directly digested and absorbed by the gastrointestinal tract. Therefore, dietary glutathione effectively enhances the antioxidant levels in tumbler pigeons, which is also one of the significant reasons for the remarkable increase in GSH-Px activity observed in the experimental group fed with *P. oleracea* [11,44,45]. Furthermore, the phenolic and flavonoid compounds found in *P. oleracea* contain highly reactive hydrogen atoms within their hydroxyl groups. These atoms can interact with free radicals, thereby interrupting the chain reactions of free radicals and enhancing the overall antioxidant capacity of living organisms [46,47].

In summary, *P. oleracea* is of great significance in influencing the metabolic activities of cells and their resistance and adaptation to various endogenous or exogenous stressor sources, enhancing the activity of antioxidant enzymes in the body, thereby improving the athletic performance of tumbler pigeons, enabling them to maintain a more sustained athletic state, alleviating athletic stress and fatigue, and achieving outstanding competitive results.

### 4.3. Effects of P. oleracea Addition in Health Care Sand on the Microbial Community Composition in Excreta of Tumbler Pigeons

The structural composition of gut microbiota within animal organisms can directly or indirectly influence multiple physiological functions of the host, including nutrient utilization, nutrient supply to the intestinal epithelium, defense against pathogenic invasion, and the development and activity of the intestinal immune system. This plays a crucial role in intestinal health and the overall health status of the host [48]. Similarly, various factors such as host genetics, lifestyle, disease, and drug therapy can in turn influence the structure and abundance of the gut microbiota [49]. The two maintain an inseparable, mutually dependent, and co-evolving symbiotic relationship [50]. Wang et al. [32] Adding *P. oleracea* L. to the diet of Sanhuang chickens significantly increased the abundance of beneficial bacteria such as Lactobacillus in the intestinal tract, while reducing the relative abundance of harmful bacteria related to diseases, such as Escherichia and Shigella of the Enterobacteriaceae family. This, in turn, regulated the internal environment of the intestinal flora, promoted carbohydrate metabolism, and improved growth performance. Research on the alleviation of carbon tetrachloride-induced acute liver injury by *P. oleracea* L. regulating the intestinal microbiota of mice has shown that *P. oleracea* polysaccharides significantly increase and decrease the abundance of Bacteroidetes and Proteobacteria in the intestinal microbiota of mice, alleviate inflammatory responses, protect the integrity of the intestinal barrier, and also activate the glycoaminoglycan degradation (GAG degradation) pathway, promoting the repair of liver cell damage Thereby assisting in the prevention and protection of liver function [51].

This study, based on Venn diagrams and OTU-level alpha diversity analysis, indicates that there are no statistically significant differences between groups. This suggests that the addition of *P. oleracea* to the health care sand does not affect the microbial community structure in the excreta of tumbler pigeons. Phylum-level analysis revealed a trend toward increased relative abundance of the Actinobacteria phylum in the intestines of pigeons added with *P. oleracea*, while simultaneously reducing the abundance of the Proteobacteria phylum. Actinobacteria and Proteobacteria are dominant phyla in the avian digestive tract [52]. Research indicates that the Actinobacteria phylum plays a crucial role in maintaining overall microbial structure and promoting host growth [53]. Actinomycetes can degrade polar starch, cellulose, and hemicellulose in feed that are difficult to be digested and utilized by the gastrointestinal tract of the body by producing abundant enzymes such as glycoside hydrolase and cellulase [54], thereby improving the efficiency of the host’s digestion and absorption of nutrients and enhancing production performance. In addition, actinomycetes are also a key source of natural bactericins and bioactive compounds, and are of great significance in many aspects such as supporting the development of the immune system, synthesizing essential vitamins for organisms, improving metabolic health, maintaining the ecological balance of intestinal microorganisms, and inhibiting pathogenic bacteria [55,56,57]. The abundance level of Proteobacteria is usually used as a key biomarker for measuring the balance of intestinal flora and the health status of livestock and poultry. It contains most of the pathogenic bacteria that have a negative impact on the host’s health, such as pathogenic Escherichia coli, Brucella, Salmonella, Helicobacter pylori, etc. [58]. These pathogenic bacteria will cause metabolic disorders, flora imbalance, diarrhea and inflammatory responses in the body by colonizing on the gastrointestinal mucosa. In summary, *P. oleracea* addition may inhibit the growth of pathogenic microorganisms by promoting the proliferation of beneficial bacteria, improve the composition of intestinal flora, and regulate the phenomenon of intestinal microecological disorder caused by long-term exercise training or high-intensity competitions, thereby enhancing intestinal physiological health, nutrient absorption and anti-inflammatory capacity.

Family analysis indicates that *P. oleracea* addition reduced the relative abundance of Chitinophagaceae in the gut microbiota of tumbler pigeons, while Nitrosotaleaceae showed an increasing trend. The Chitinophagaceae family belongs to the Bacteroidetes phylum. The chitinase they secrete efficiently degrades chitin, a complex polysaccharide widely present in the exoskeletons of crustaceans, insects, and fungal cell walls. The composition of the health grit used in this study contains a high level of shell powder (33.5%). *P. oleracea* itself is rich in various bioactive compounds, such as norepinephrine, flavonoids, polysaccharides, and omega-3 fatty acids, which provide specific food sources for gut microbiota. These substances may be more readily and efficiently utilized by the host itself or other beneficial bacteria [59], thereby reducing dependence on bacteria requiring specialized degradation of complex fibers- chitin-degrading bacteria (Chitinibacter family). This could be a key reason why the Chitinibacter family abundance was higher in the control group than in the *P. oleracea* treatment group in this study. Adding *P. oleracea* can increase the relative abundance of the Nitrosotaleaceae family. Since this bacterial family carries the ammonia monooxygenase (amoA) gene [60], it improves gut health by converting toxic ammonia into less harmful nitrate. This reduction in ammonia toxicity may help protect the integrity of the gut barrier, providing a physiological basis for enhancing racing pigeon performance. However, the mechanism of action of Nitrosotaleaceae in the gastrointestinal tract of livestock and poultry still requires further in-depth exploration.

LEfSe analysis showed that g_Pseudoflavitalea was the dominant flora in the CON group of tumbler pigeons, while the TRT1 group was mainly enriched with *s_Acidobacteria_bacterium*. The genus Acidobacteria belongs to the phylum Acidobacteria and is typically regarded as a beneficial or “symbiotic” member of the gut microbiota. These bacteria are capable of degrading plant-derived polymers and other biopolymers [61], particularly chitin, cellulose, hemicellulose, and xylan. The short-chain fatty acids produced during this degradation serve as an important energy source for both the gut microbiota and the host’s intestinal epithelial cells. Furthermore, short-chain fatty acids can promote the growth and proliferation of beneficial bacteria in the gastrointestinal tract by lowering intestinal pH, reducing pathogen adhesion, and alleviating inflammatory responses. This collectively contributes to maintaining gastrointestinal health [62].

To better elucidate the effects of the microbiota on host physiology, this study further employed PICRUSt2 to predict the metabolic functions of the gut microbiota. Adding *P. oleracea* had varying degrees of impact on the digestive physiology of tumbler pigeons. The functional enrichment of purslane-added pigeon droppings primarily manifests in Ribosome biogenesis, Pyruvate metabolism, Carbon fixation pathways in prokaryotes, and Quorum sensing. These findings indicate purslane’s crucial role in enhancing energy utilization and nutrient absorption, emphasizing its positive impact on both the gut microbiota and the host. This aligns with the biological functions associated with *P. oleracea* [63]. It can be concluded that *P. oleracea* not only alters the composition and structure of dominant microbial communities in the intestines of racing pigeons but also enriches the functional diversity of microorganisms, thereby playing a positive role in maintaining the intestinal environment.

### 4.4. Effects of P. oleracea Addition in Health Care Sand on the Metabolites in Excreta of Tumbler Pigeons

The intestinal microbiota can produce a large number of metabolic products, such as amino acids, organic acids, nucleotides, peptides, lipids, carbohydrates and cofactors and other small molecule substances. Under nutritional intervention conditions, that is, specific feed raw materials or additives, these metabolites reflect the results of the intestinal microbiota’s uptake, digestion and utilization of nutrients [64,65]. The process that simultaneously causes corresponding changes in endogenous metabolites within the animal’s body is of great significance for in-depth and comprehensive evaluation of the specific metabolic pathways, mechanisms, and state changes of nutrients within the body [66]. To this end, this study employed LC-MS metabolomics analysis to further evaluate the effects of *P. oleracea* on metabolites in the excreta of tumbler pigeons. Results indicate that compared with the CON group, *Portulaca olerace* addition significantly increased the levels of differential metabolites including agmatine, pyropheophorbide-a, *N*-acetylmuramate, thiamine, 2′-deoxycytidine, DL-mannitol, and *N*-acetylcitrulline, while markedly decreasing the levels of indoleacetic acid and 4-hydroxyaniline.

Agmatine is a neuromodulator produced from endogenous L-arginine via arginine decarboxylase. It exerts neuroprotective effects in brain dysfunction and stress response systems, while also exhibiting anti-inflammatory, anti-apoptotic, and antioxidant properties [67,68]. The research conducted by Bahremand et al. [69] indicates that agmatine may significantly enhance the anticonvulsant effects against swimming stress in mice by mediating the nitric oxide signaling pathway. During high-speed somersaults and complex maneuvers, tumbler pigeons may potentially experience transient neurological stress due to intense physical exertion and constant changes in head position. The results of this study indicate that adding *P. oleracea* can significantly increase the concentration of agmatine metabolites. This suggests that this strain, by regulating the gut–brain axis, can help pigeons recover from exercise-induced brain dysfunction but also enhance their anti-inflammatory and stress-resistance capabilities. Pyropheophorbide-a, a porphyrin-based antimicrobial substance, generates reactive oxygen species that act on pathogenic microorganisms, inhibiting their growth or reproduction [70]. In this study, the TRT1 group exhibited an increased level of the gut microbiota metabolite pyropheophorbide-a in tumbler pigeons. It is hypothesized that *P. oleracea* may inhibit the growth and colonization of certain pathogenic bacteria by elevating the concentration of pyropheophorbide-a, thereby contributing to the maintenance of intestinal health. Under certain conditions, indolelactic acid helps strengthen connections between intestinal epithelial cells [71]. Furthermore, research reports indicate that the abundance of Akkermansia muciniphila is associated with the production of indole and its derivatives [72,73]. The results of this study indicate that, compared to the CON group, the levels of the gut microbiota metabolite indolelactic acid were significantly reduced in both the TRT1 and TRT2 groups. This suggests that *P. oleracea* may alter the structure and composition of gut microbial communities, potentially by decreasing the relative abundance of Akkermansia muciniphila species, thereby influencing the production of indolelactic acid. The reasons for this result still need further in-depth research. *N*-acetylmuramate is a crucial component of bacterial cell wall peptidoglycan. An increase in its levels directly reflects the heightened activity of bacterial growth, death, and lysis processes within the gut [74]. It is hypothesized that addition with *P. oleracea* in this study may accelerate metabolic rates and promote the physiological health of the host. The main functions of 4-hydroxyaniline are reflected in its toxic effects and metabolic processes. 4-Hydroxyaniline is metabolized and oxidized in liver tissue into an intermediate of benzoquinone imine, which can directly damage liver cells and may lead to liver cell necrosis and liver failure. In addition, 4-hydroxyaniline binds to the antioxidant glutathione, causing its depletion and further exacerbating oxidative stress damage [75]. In this study, the TRT1 and TRT2 groups exhibited significantly reduced relative concentrations of 4-hydroxyaniline. This indicates that *P. oleracea*, through its antioxidant and hepatoprotective functions, alleviates metabolic stress in tumbler pigeons, reduces the accumulation of toxic metabolites, and thereby exerts positive health-promoting effects. Thiamine, also known as vitamin B1, typically participates in nutritional metabolic processes within animal organisms in the form of coenzymes. Thiamine plays a crucial role in numerous beneficial aspects, including protein biosynthesis, amino acid metabolism, energy metabolism, and neural regulation [76]. The results of this study indicate that *P. oleracea* can significantly increase the thiamine content in tumbler pigeons excreta metabolites, revealing its important role in promoting energy supply, neuromuscular coordination, flight endurance, and overall health. 2′-Deoxycytidinec, as a direct raw material for DNA synthesis, is closely related to cell proliferation, tissue repair and DNA synthesis activities [77]. DL-Mannitol can regulate plasma osmotic pressure, facilitating the transfer of water from tissue cells into the bloodstream, thereby achieving a balance in osmotic pressure. Additionally, it possesses hydroxyl radical scavenging properties that help prevent oxidative stress damage [78]. For racing pigeons facing dehydration and metabolic stress during high-intensity training and competitions, *P. oleracea* may play a crucial role by influencing osmotic regulation and cellular protective mechanisms within the pigeon’s body. *N*-Acetylcitrulline is the acetylated form of citrulline. Upon entering the body, it undergoes deacetylation to convert back into citrulline. Can dilates blood vessels and regulates blood pressure, thereby enhancing oxygen and nutrient delivery [79]. The non-targeted metabolomic analysis revealed a significant increase in *N*-acetylcitrulline in the excreta samples of the TRT2 group, indicating that *P. oleracea* may be able to improve the blood circulation and nitrogen metabolism of tumbler pigeons to enhance their flight performance and recovery ability. In this study, metabolites significantly enriched after *P. oleracea* addition were observed in metabolic pathways including Tryptophan metabolism, Drug metabolism-cytochrome P450, Metabolic pathways, Porphyrin and chlorophyll metabolism, and ABC transporters. This reveals purslane’s significant role in regulating energy supply, nervous system development, liver detoxification capacity, and protein and amino acid biosynthesis, among other beneficial functions. Consistent with the microbial functional prediction results mentioned above, this provides deeper evidence for the intrinsic relationship between gut microbiota and their metabolites. This suggests that *P. oleracea* may improve gut health by regulating these key metabolites and metabolic pathways, thereby modulating the physiological functions and metabolic state of the intestinal tissue, ultimately enhancing the body’s digestion and absorption of nutrients and improving host health.

## 5. Conclusions

In summary, adding 1% *P. oleracea* to health care sand significantly enhanced nutrient digestibility, improved systemic antioxidant capacity, and modulated the gut microbiota and metabolome in tumbler pigeons. Notably, these beneficial effects were more pronounced at the 1.00% inclusion level compared to the 0.75% level, suggesting a dose-dependent response. These findings demonstrate that *P. oleracea* can serve as an effective natural feed additive to support athletic performance and metabolic health in racing pigeons, but its efficacy is contingent upon the optimal inclusion rate. Incorporating 1.00% *P. oleracea* powder into health care sand offers a feasible and sustainable nutritional strategy for improving exercise recovery and nutrient utilization in pigeons engaged in high-intensity flight training. However, this study has several limitations. The sample size, though consistent with poultry nutrition studies, was relatively small. In addition, the dose gradient was limited to two treatment levels (0.75% and 1.00%), and higher supplementation rates were not explored. Future research should include histological examinations of intestinal tissues, broader dose-ranging trials, and molecular analyses to further elucidate the mechanisms underlying the observed effects and to precisely determine the optimal dietary incorporation rate.

## Figures and Tables

**Table 1:** Nutrient content of experimental diets for pigeons.

Composition	(Content, g/kg)	Nutrient Index	Nutritional Status
Corn	650	DM (%)	91.96
Soybeanmeal	200	OM (%)	96.18
Secondary powder	50	ME MJ/kg	10.87
Cotton protein	50	CP (%)	18.34
Soybean oil	10	EE (%)	2.00
Limestone (0~2 mm)	10	CF (%)	3.05
Limestone (2~4 mm)	10	Ca (%)	0.94
2% Premix	20	P (%)	0.58
Total	1000	Lys (%)	0.86
		Met (%)	0.44

Note: The premix provides the following per kilogram of the diet: Vitamin A 5330 IU; Vitamin D3 1300 IU; Vitamin E 30 mg; Vitamin K3 3.10 mg; Vitamin B1 2.00 mg; Vitamin B2 7.00 mg; Biotin 0.10 mg; Folic acid 1.30 mg; Niacin 37 mg; Calcium pantothenate 2300 mg; Lysine 5400 mg; Methionine 2300 mg. DM, OM, GE, ME, CP, EE, and CF are measured values, while the rest are calculated values.

**Table 2:** Composition of health care sand (%).

Composition	CON	TRT1	TRT2
Shell powder	33.5	33.5	33.5
Red clay	24.8	24.8	24.8
Maifan stone	13.6	13.6	13.6
Red peck soil	8.5	8.5	8.5
Zeolite powder	11.4	10.65	10.4
Sea salt	4.2	4.2	4.2
Charcoal	3	3	3
Mycotoxin adsorbent	1	1	1
*Portulaca oleracea*	0	0.75	1

**Table 3:** Effects of *P. oleracea* addition in health care sand on apparent nutrient digestibility and metabolism in tumbler pigeons.

Items	CON	TRT1	TRT2
DM%	72.30 ± 2.05 ^Aa^	64.74 ± 1.74 ^Bb^	70.24 ± 4.67 ^ABa^
OM%	77.44 ± 1.40 ^ab^	76.01 ± 0.74 ^b^	79.24 ± 2.36 ^a^
CP%	41.16 ± 4.01 ^ABb^	37.79 ± 4.41 ^Bb^	48.04 ± 3.49 ^Aa^
GE%	79.06 ± 1.02 ^A^	76.33 ± 0.69 ^B^	79.32 ± 2.04 ^A^
ME MJ/kg	10.87 ± 0.14 ^A^	10.49 ± 0.09 ^B^	10.90 ± 0.28 ^A^
EE%	78.90 ± 2.37	80.30 ± 11.46	78.65 ± 4.40

Note: DM, dry matter; OM, organic matter; CP, crude protein; ME, metabolizable energy; GE, gross energy; EE, ether extract. Values within the same row with no superscript letters or identical superscripts indicate no significant difference (*p* > 0.05). Values with different lowercase letters indicate significant differences (^a, b^: *p* < 0.05), and those with different uppercase letters indicate highly significant differences (^A, B^: *p* < 0.01). CON group: basal diet +4 g health care sand; TRT1 group: basal diet +4 g health care sand containing 0.75% *P. oleracea*; TRT2 group: basal diet +4 g health care sand containing 1.00% *P. oleracea*. Data are presented as mean ± standard deviation (*n* = 10).

## Data Availability

The data that support the findings of this study are available from the corresponding author upon reasonable request.

## Supplementary Materials

The following supporting information can be downloaded at: https://www.mdpi.com/article/10.3390/ani15223349/s1, Table S1: Effects of *Portulaca oleracea* addition in health care sand on apparent nutrient digestibility and metabolism in tumbler pigeons; Table S2: Effects of *Portulaca oleraceae* addition in health care sand on serum biochemical and antioxidant capacity in tumbler pigeons; Table S3: Relative abundance of microbial communities at the phylum level; Table S4: Relative abundance of microbial communities at the family level; Table S5: Effects of *Portulaca oleracea* addition in health care sand on metabolites in tumbler pigeons.

## References

[1] Entrikin R.K., Bryant S.H. (1974). Tumbling in pigeons. Nature.

[2] STUDENS (1871). The Genesis of Species. Nature.

[3] Li X., Guo X., Li H., Liu J., Lin J., Zheng S., Ke L. (2025). Effects of different metabolizable energy levels on apparent nutrient digestibility and metabolism, blood biochemical indicators, and fecal flora diversity in racing pigeons undergoing exercise training. Front. Microbiol..

[4] Ning C., Bu W., Meng X., Li H., Zhang X., Tang Y., Hu F., Wang S., Tan C., Guo C. (2025). Study on anti-fatigue effect and mechanism of iron source combined with *Angelica sinensis* and *Agrimonia pilosa* on pigeons under exercise stress. Poult. Sci..

[5] Kastelic M., Pšeničnik I., Gračner G.G., Kadunc N.Č., Knific R.L., Slavec B., Krapež U., Rataj A.V., Rojs O.Z., Pulko B. (2021). Health status and stress in different categories of racing pigeons. Animals.

[6] Yu Q.P., Feng D.Y., Xia M.H., He X.J., Liu Y.H., Tan H.Z., Zou S.G., Ou X.H., Zheng T., Cao Y. (2017). Effects of a traditional Chinese medicine formula supplementation on growth performance, carcass characteristics, meat quality and fatty acid profiles of finishing pigs. Livest. Sci..

[7] Abdallah A., Zhang P., Zhong Q., Sun Z. (2019). Application of traditional Chinese herbal medicine by-products as dietary feed supplements and antibiotic replacements in animal production. Curr. Drug Metab..

[8] Gorske S.F., Rhodes A.M., Hopen H.J. (1979). A numerical taxonomic study of *Portulaca oleracea*. Weed Sci..

[9] Li K., Xia T., Jiang Y., Wang N., Lai L., Xu S., Yue X., Xin H. (2024). A review on ethnopharmacology, phytochemistry, pharmacology and potential uses of *Portulaca oleracea* L. J. Ethnopharmacol..

[10] Li Y., Xiao L., Yan H., Wu M., Hao X., Liu H. (2024). Nutritional values, bioactive compounds and health benefits of purslane (*Portulaca oleracea* L.): A comprehensive review. Food Sci. Hum. Wellness.

[11] Iranshahy M., Javadi B., Iranshahi M., Jahanbakhsh S.P., Mahyari S., Hassani F.V., Karimi G. (2017). A review of traditional uses, phytochemistry and pharmacology of *Portulaca oleracea* L. J. Ethnopharmacol..

[12] Jalali J., Ghasemzadeh Rahbardar M. (2022). Ameliorative effects of *Portulaca oleracea* L. (purslane) on the metabolic syndrome: A review. J. Ethnopharmacol..

[13] Liu J., Jiu J., Zhang X., Sun J., Ying X. (2025). Four alkaloids from *Portulaca oleracea* L. and their anti-inflammatory. Nat. Prod. Res..

[14] Du Y.K., Liu J., Li X.M., Pan F.F., Wen Z.G., Zhang T.C., Yang P.L. (2017). Flavonoids extract from *Portulaca oleracea* L. induce *Staphylococcus aureus* death by apoptosis-like pathway. Int. J. Food Prop..

[15] Bai Y., Zang X., Ma J., Xu G. (2016). Anti-diabetic effect of *Portulaca oleracea* L. Polysaccharideandits mechanism in diabetic rats. Int. J. Mol. Sci..

[16] Zhou X., Li Y., Li T., Cao J., Guan Z., Xu T., Jia G., Ma G., Zhao R. (2023). *Portulaca oleracea* L. polysaccharide inhibits porcine rotavirus in vitro. Animals.

[17] Tian X. (2022). Protective Effect and Mechanism of *Portulaca oleracea* L. Dietary Fiber on Cadmium Exposure in Mice. Master’s Thesis.

[18] (1994). Method for Determination of Crude Protein in Feed.

[19] (2006). Determination of Crude Fat in Feeds.

[20] Van Soest P.J., Robertson J.B., Lewis B.A. (1991). Methods for dietary fiber, neutral detergent fiber, and nonstarch polysaccharides in relation to animal nutrition. J. Dairy Sci..

[21] (2002). Determination of Total Phosphorus in Feed (Spectrophotometric Method).

[22] Oliveira D.R., Lopes A.C.A., Pereira R.A., Cardoso P.G., Duarte W.F. (2019). Selection of potentially probiotic *Kluyveromyces lactis* for the fermentation of cheese whey–based beverage. Ann Microbiol..

[23] Shivamathi C.S., Gunaseelan S., Soosai M.R., Vignesh N.S., Varalakshmi P., Kumar R.S., Karthikumar S., Kumar R.V., Baskar R., Rigby S.P. (2022). Process optimization and characterization of pectin derived from underexploited pineapple peel biowaste as a value-added product. Food Hydrocoll..

[24] Shraim A.M., Ahmed T.A., Rahman M.M., Hijji Y.M. (2021). Determination of total flavonoid content by aluminum chloride assay: A critical evaluation. LWT.

[25] (2014). Determination of Moisture Content in Feed.

[26] (2007). Determination of Crude Ash Content in Feed.

[27] (2008). Determination of Moisture and Volatile Matter Content in Oilseeds.

[28] Huang X., He L., Ma J., Li Y., Li J., Zang C., Hou M., Li X. (2025). Ellagic acid on milk production performance, blood and milk hormones, antioxidant capacity and fecal microbial communities in lactating Yili mares. Front. Microbiol..

[29] Cheng X., Tan Y., Li H., Huang J., Zhao D., Zhang Z., Yi M., Zhu L., Hui S., Yang J. (2022). Fecal 16S rRNA sequencing and multi-compartment metabolomics revealed gut microbiota and metabolites interactions in APP/PS1 mice. Comput. Biol. Med..

[30] Li X., Zheng S., Li H., Liu J., Yang F., Zhao X., Liang Y. (2025). 16S rRNA sequencing and metabolomics to analyze correlation between fecal flora and metabolites of squabs and parent pigeons. Animals.

[31] Abd El-Hack M.E., Alabdali A.Y.M., Aldhalmi A.K., Reda F.M., Bassiony S.S., Selim S., El-Saadony M.T., Alagawany M. (2022). Impacts of Purslane (*Portulaca oleracea*) extract supplementation on growing japanese quails’ growth, carcass traits, blood indices, nutrients digestibility and gut microbiota. Poult. Sci..

[32] Wang C., Liu Q., Ye F., Tang H., Xiong Y., Wu Y., Wang L., Feng X., Zhang S., Wan Y. (2021). Dietary purslane (*Portulaca oleracea* L.) promotes the growth performance of broilers by modulation of gut microbiota. AMB Express.

[33] Han X., Yang Y., Zhang N., Liu H., Zhang Y., Du X., He X., Zhao X. (2025). Effects of *Portulaca oleracea* extract on growth performance, antioxidant capacity and intestinal barrier of Wenchang chickens. Feed Res..

[34] Ning K., Shi C., Chi Y.Y., Zhou Y.F., Zheng W., Duan Y., Tong W., Xie Q., Xiang H. (2024). *Portulaca oleracea* L. polysaccharide alleviates dextran sulfate sodium-induced ulcerative colitis by regulating intestinal homeostasis. Int. J. Biol. Macromol..

[35] Fu Q., Zhou S., Yu M., Lu Y., He G., Huang X., Huang Y. (2022). *Portulaca oleracea* Polysaccharides Modulate Intestinal Microflora in Aged Rats in Vitro. Front. Microbiol..

[36] Habibian M., Sadeghi G., Karimi A. (2019). Comparative effects of powder, aqueous and methanolic extracts of purslane (*Portulaca oleracea* L.) on growth performance, antioxidant status, abdominal fat deposition and plasma lipids in broiler chickens. Anim. Prod. Sci..

[37] Eidi A., Mortazavi P., Moghadam J.Z., Mardani P.M. (2015). Hepatoprotective effects of *Portulaca oleracea* extract against CCl4-induced damage in rats. Pharm. Biol..

[38] Westerblad H., Allen D.G., Lännergren J. (2002). Muscle fatigue: Lactic acid or inorganic phosphate the major cause?. Physiology.

[39] Spriet L.L., Howlett R.A., Heigenhauser G.J.F. (2000). An enzymatic approach to lactate production in human skeletal muscle during exercise. Med. Sci. Sports Exerc..

[40] Chen X., He Z., Wang Z., Li H. (2023). The effect of the purslane polyphenols on the structure of rabbit meat myofibrillar protein under malondialdehyde-induced oxidative stress. J. Food Sci..

[41] Margonis K., Fatouros I.G., Jamurtas A.Z., Nikolaidis M.G., Douroudos I., Chatzinikolaou A., Mitrakou A., Mastorakos G., Papassotiriou I., Taxildaris K. (2007). Oxidative stress biomarkers responses to physical overtraining: Implications for diagnosis. Free Radic. Biol. Med..

[42] Xu L., Gao G., Zhou Z., Wei Z., Sun W., Li Y., Jiang X., Gu J., Li X., Pi Y. (2024). Fermented purslane (*Portulaca oleracea* L.) supplementation enhances growth and immune function parallel to the regulation of gut microbial butyrate production in weaned piglets. Microorganisms.

[43] Dahran N., Alotaibi B.S., Abd-Elhakim Y.M., Mohamed A.A.-R., Ibrahim R.E., Metwally M.M.M., Khamis T., Eskandrani A.A., Alosaimi M.E., Aly M.Y.M. (2025). Dietary purslane (*Portulaca oleracea* L.) leaf powder maintains growth and intestinal health in Oreochromis niloticus under chronic water-borne cadmium exposure by strengthening the gut barriers, modulating the intestinal nutrient transporters, and relieving oxidative stress. Fish Physiol. Biochem..

[44] Simopoulos A.P., Norman H.A., Gillaspy J.E., Duke J.A. (1992). Common purslane: A source of omega-3 fatty acids and antioxidants. J. Am. Coll. Nutr..

[45] Simopoulos A.P. (2001). The Mediterranean diets: What is so special about the diet of Greece? The scientific evidence. J. Nutr..

[46] Erkan N. (2012). Antioxidant activity and phenolic compounds of fractions from *Portulaca oleracea* L. Food Chem..

[47] Duan Y., Ying Z., Zhang M., Ying X., Yang G. (2020). Two new homoisoflavones from *Portulaca oleracea* L. and their activities. Nat. Prod. Res..

[48] Barrett K.E., Wu G.D. (2017). Influence of the Microbiota on Host Physiology—Moving beyond the Gut. J. Physiol..

[49] Bonder M.J., Kurilshikov A., Tigchelaar E.F., Mujagic Z., Imhann F., Vila A.V., Deelen P., Vatanen T., Schirmer M., Smeekens S.P. (2016). The Effect of host genetics on the gut microbiome. Nat. Genet..

[50] Broom L.J. (2019). Host–microbe interactions and gut health in poultry—Focus on innate responses. Microorganisms.

[51] Li J., Chen Y., Zhang S., Zhao Y., Gao D., Xing J., Cao Y., Xu G. (2025). Purslane (*Portulaca oleracea* L.) polysaccharide attenuates carbon tetrachloride-induced acute liver injury by modulating the gut microbiota in mice. Genomics.

[52] Kers J.G., Velkers F.C., Fischer E.A.J., Hermes G.D.A., Stegeman J.A., Smidt H. (2018). Host and environmental factors affecting the intestinal microbiota in chickens. Front. Microbiol..

[53] Binda C., Lopetuso L.R., Rizzatti G., Gibiino G., Cennamo V., Gasbarrini A. (2018). Actinobacteria: A relevant minority for the maintenance of gut homeostasis. Dig. Liver Dis..

[54] Barka E.A., Vatsa P., Sanchez L., Gaveau-Vaillant N., Jacquard C., Klenk H.-P., Clément C., Ouhdouch Y., Wezel G.P. (2016). Taxonomy, physiology, and natural products of *Actinobacteria*. Microbiol. Mol. Biol. Rev..

[55] Hui M.L., Tan L.T., Letchumanan V., He Y.W., Fang C.M., Chan K.G., Law J.W., Lee L.H. (2021). The extremophilic actinobacteria: From microbes to medicine. Antibiotics.

[56] Trosvik P., de Muinck E.J. (2015). Ecology of bacteria in the human gastrointestinal tract—Identification of keystone and foundation taxa. Microbiome.

[57] Pinheiro G.L., Correa R.F., Cunha R.S., Cardoso A.M., Chaia C., Clementino M.M., Garcia E.S., de Souza W., Frasés S. (2015). Isolation of aerobic cultivable cellulolytic bacteria from different regions of the gastrointestinal tract of giant land snail Achatina fulica. Front. Microbiol..

[58] Rizzatti G., Lopetuso L.R., Gibiino G., Binda C., Gasbarrini A. (2017). Proteobacteria: A common factor in human diseases. BioMed Res. Int..

[59] Li Z., Chu T., Sun X., Zhuang S., Hou D., Zhang Z., Sun J., Liu Y., Li J., Bian Y. (2025). Polyphenols-rich *Portulaca oleracea* L. (Purslane) alleviates ulcerative colitis through restiring the intestinal barrier, gut microbiota and metabolites. Food Chem..

[60] Zhao J., Wang B., Zhou X., Alam M.S., Fan J., Guo Z., Zhang H., Gubry-Rangin C., Zhongjun J. (2022). Long-Term Adaptation of Acidophilic Archaeal Ammonia Oxidisers Following Different Soil Fertilisation Histories. Microb. Ecol..

[61] Sikorski J., Baumgartner V., Birkhofer K., Boeddinghaus R.S., Bunk B., Fischer M., Fösel B.U., Friedrich M.W., Göker M., Hölzel N. (2022). The evolution of ecological diversity in Acidobacteria. Front. Microbiol..

[62] Kielak A.M., Barreto C.C., Kowalchuk G.A., van Veen J.A., Kuramae E.E. (2016). The ecology of Acidobacteria: Moving beyond genes and genomes. Front. Microbiol..

[63] Neto L.J.V., de Araujo M.R., Junior R.C.M., Machado N.M., Joshi R.K., Buglio D.d.S., Lamas C.B., Direito R., Laurindo L.F., Tanaka M. (2024). Investigating the neuroprotective and cognitive-enhancing effects of *Bacopa monnieri*: A systematic review focused on inflammation, oxidative stress, mitochondrial dysfunction, and apoptosis. Antioxidants.

[64] Tanes C., Hu W., Friedman E., Hecht A., Daniel S., Clish C., Lewis J.D., Wu G.D., Bittinger K. (2025). Distinguishing Diet- and Microbe-Derived Metabolites in the Human Gut. Microbiome.

[65] Wang H., Xu R., Zhang H., Su Y., Zhu W. (2020). Swine Gut Microbiota and Its Interaction with Host Nutrient Metabolism. Anim. Nutr..

[66] Kortesniemi M., Noerman S., Kårlund A., Raita J., Meuronen T., Koistinen V., Landberg R., Hanhineva K. (2023). Nutritional metabolomics: Recent developments and future needs. Curr. Opin. Chem. Biol..

[67] Chang J.Y., Kim J., Kosonen R., Kim J.Y., Lee J.E. (2025). The Role of Agmatine in modulating autophagy under neuroinflammatory conditions induced by metabolic alteration in mouse brain. Exp. Neurobiol..

[68] Cobos-Puc L.E., Aguayo-Morales H. (2025). Agmatine mitigates diabetes-related memory loss in female mice by targeting i_2_/i_3_ imidazoline receptors and enhancing brain antioxidant defenses. Antioxidants.

[69] Bahremand T., Payandemehr P., Riazi K., Noorian A.R., Payandemehr B., Sharifzadeh M., Dehpour A.R. (2018). Modulation of the anticonvulsant effect of swim stress by agmatine. Epilepsy Behav..

[70] Nakano M., Sakamoto T., Itoh Y., Kitano Y., Tsukakoshi K., Bono H., Tabunoki H. (2025). The metabolic ability of swallowtails results in the production of bioactive substances from plant components. PLoS ONE.

[71] Qiu J., Heller J.J., Guo X., Chen Z.E., Fish K., Fu Y.-X., Zhou L. (2012). The aryl hydrocarbon receptor regulates gut immunity through modulation of innate lymphoid cells. Immunity.

[72] Gu Z., Pei W., Shen Y., Wang L., Zhu J., Zhang Y., Fan S., Wu Q., Li L., Zhang Z. (2021). *Akkermansia muciniphila* and its outer protein Amuc_1100 regulates tryptophan metabolism in colitis. Food Funct..

[73] Yin J., Song Y., Hu Y., Wang Y., Zhang B., Wang J., Ji X., Wang S. (2021). Dose-dependent beneficial effects of tryptophan and its derived metabolites on Akkermansia in vitro: A preliminary prospective study. Microorganisms.

[74] Hu M., Xu Y., Wang Y., Huang Z., Wang L., Zeng F., Qiu B., Liu Z., Yuan P., Wan Y. (2025). Gut microbial-derived N-acetylmuramic acid alleviates colorectal cancer via the AKT1 pathway. Gut.

[75] Fu X., Chen T.S., Ray M.B., Nagasawa H.T., Williams W.M. (2004). *p*-Aminophenol-induced hepatotoxicity in hamsters: Role of glutathione. J. Biochem. Mol. Toxicol..

[76] Bozic I., Lavrnja I. (2023). Thiamine and benfotiamine: Focus on their therapeutic potential. Heliyon.

[77] Rokita S.E., Yang J., Pande P., Greenberg W.A. (1997). Quinone Methide Alkylation of Deoxycytidine. J. Org. Chem..

[78] Reiterer C., Hu K., Sljivic S., Falkner von Sonnenburg M., Fleischmann E., Kabon B. (2021). The effect of mannitol on oxidation-reduction potential in patients undergoing deceased donor renal transplantation—A randomized controlled trial. Acta Anaesthesiol. Scand..

[79] Figueroa A., Jaime S.J., Morita M., Gonzales J.U., Moinard C. (2020). L-Citrulline Supports Vascular and Muscular Benefits of Exercise Training in Older Adults. Exerc. Sport Sci. Rev..

