# Supplementary material for: Effect of Portulaca oleracea Addition in Health Care Sand on Apparent Nutrient Digestibility, Serum Parameters, and Excreta Microbiota Metabolism in Tumbler Pigeons
Source: Animals (Basel). 2025 Nov 20;15(22):3349. doi: 10.3390/ani15223349 (PMC12649108; doi:10.3390/ani15223349)
Supplement: Supplementary file 1 [file animals-15-03349-s001.zip › animals-3987814-supplementary.pdf]

Table S1. Effects of *Portulaca oleracea* supplementation in health care sand on apparent nutrient digestibility and metabolism in tumbler pigeons

	DM %	OM %	CP %	ME MJ/kg	GE %	EE %
CON	69.55909561	75.95310282	35.06944886	10.69472978	77.80177303	76.62189062
CON	74.03937301	78.68967945	44.48910799	11.00831831	80.08305965	76.24497254
CON	73.96895548	78.39952348	44.64471852	10.94440471	79.61810248	81.24871103
CON	73.24932709	78.26590999	42.19326087	10.95282077	79.67932741	80.98992068
CON	70.67539937	75.88087849	39.40768692	10.73918149	78.12514929	79.41781161
TRT1	63.26074209	76.08235144	37.36591041	10.50903002	76.45084872	79.85328929
TRT1	64.99180861	76.71515354	37.42907641	10.64124954	77.41271623	85.28600804
TRT1	66.81956665	76.11312039	34.9618298	10.47752154	76.2216316	95.36981156
TRT1	65.94190742	76.37949622	45.20324984	10.44215047	75.96431494	64.11553534
TRT1	62.70739615	74.76684852	33.99512795	10.39091047	75.59155532	76.88827351
TRT2	64.74161914	76.49077087	44.72640331	10.937796	79.57002556	71.67643928
TRT2	67.92051835	78.83827275	44.91221058	10.69376336	77.79474253	79.76780383
TRT2	69.47633283	77.74857344	49.65844919	10.56922276	76.88873745	77.37820026
TRT2	77.18217221	82.45564353	53.06490986	11.27518346	82.02444408	82.69661871
TRT2	71.90241767	80.654345	47.81856204	11.04173374	80.3261494	81.73347874

Table S2. Effects of *Portulaca oleraceae* supplementation in health care sand on serum biochemical and antioxidant capacity in tumbler pigeons.

	TP	ALB	GLB	AST	ALT	ALP	TC	GLU	LAC	LDH	UA	TG	MDA	SOD	GSH-PX	T-AOC	CAT
CON	28.736	17.455	11.281	235.064	90.558	364.272	11.208	20.668	3.486	65.315	251.734	1.034	4.119	78.955	138.803	7.343	31.566
CON	26.486	16.374	10.112	145.062	68.929	373.728	11.584	22.343	2.591	53.1	261.469	1.25	3.062	81.271	146.858	7.324	35.636
CON	27.146	16.681	10.465	152.62	62.361	310.473	11.081	24.068	4.143	56.141	249.941	1.401	4.452	74.679	150.29	7.056	33.502
CON	29.223	16.092	13.131	206.068	46.098	990.83	10.21	21.057	2.003	62.407	220.444	1.092	3.277	70.776	143.018	7.129	36.904
CON	25.519	15.839	9.68	152.647	50.79	468.366	11.711	23.692	2.282	44.808	322.436	1.263	2.695	70.967	148.811	7.324	37.334
CON	28.822	17.691	11.131	139.382	40.411	335.91	12.912	25.289	2.468	48.276	246.617	0.959	2.919	80.001	152.784	7.333	39.661
CON	27.066	16.276	10.79	156.041	39.028	221.671	12.346	22.868	2.579	56.736	243.158	1.125	3.046	78.54	155.314	7.074	46.183
CON	26.334	16.204	10.13	140.052	49.337	381.523	10.607	23.857	4.199	101.288	219.151	1.355	4.508	79.163	137.422	6.685	38.317
CON	27.58	16.352	11.228	189.002	49.581	407.774	12.741	23.647	2.213	49.415	225.774	1.021	3.524	65.576	155.314	7.147	34.913
CON	29.97	16.315	13.655	190.149	42.647	509.846	9.947	22.218	2.79	55.593	146.061	1.295	3.022	73.491	144.448	8.516	37.011
TRT1	26.82	15.09	11.73	226.227	63.993	337.185	10.552	22.63	2.406	68.009	309.895	0.919	2.866	78.955	170.166	8.747	39.888
TRT1	27.807	16.498	11.309	259.531	72.096	352.461	10.419	20.443	2.655	44.075	392.338	1.275	2.367	86.067	173.504	8.217	42.943
TRT1	32.705	16.633	16.072	254.334	58.607	396.433	12.698	22.968	2.847	87.642	295.288	1.207	4.067	84.078	153.792	7.212	50.577
TRT1	29.262	17.241	12.021	170.634	51.999	514.11	11.078	21.736	2.552	45.04	293.578	1.24	2.439	76.083	165.263	8.082	40.809
TRT1	30.246	16.673	13.573	155.668	49.927	863.634	10.253	21.218	2.847	49.684	463.251	1.196	2.904	89.697	192.902	8.323	46.44
TRT1	29.51	16.885	12.625	183.692	56.967	294.071	12.1	23.617	2.082	60.958	335.246	1.159	3.277	94.894	128.083	8.113	41.158
TRT1	25.969	16.798	9.171	212.592	49.007	525.403	12.722	21.915	3.105	76.7	293.809	1.34	4.036	85.621	178.043	8.041	42.701
TRT1	27.695	14.866	12.829	198.974	50.513	325.377	9.528	21.96	2.781	67.986	343.999	1.139	2.547	86.514	189.847	7.758	41.628
TRT1	26.695	15.768	10.927	174.659	41.701	554.429	10.297	23.85	2.565	38.726	207.732	1.13	3.673	72.318	155.825	7.63	37.659
TRT1	29.599	16.985	12.614	110.786	29.069	308.341	12.32	21.28	2.249	51.09	254.464	0.905	3.218	77.509	143.018	8.769	43.551
TRT2	28.728	16.339	12.389	197.143	50.296	330.388	10.303	21.289	2.331	94.964	424.7	0.947	2.666	97.092	195.378	9.041	45.799

TRT2	30.69	15.759	14.931	168.766	39.04	551.609	9.868	22.088	1.987	64.861	297.104	1.014	2.348	98.083	187.433	8.658	45.292
TRT2	29.454	16.595	12.859	158.797	40.993	305.702	10.47	21.991	3.092	58.064	198.438	1.029	2.017	95.379	191.064	8.473	45.927
TRT2	26.22	15.602	10.618	327.853	103.074	279.416	10.801	22.354	2.392	127.025	274.69	1.299	2.012	85.621	178.043	8.78	46.183
TRT2	26.11	14.924	11.186	278.591	73.645	422.657	9.881	21.421	2.368	115.522	298.826	1.09	2.162	88.093	185.046	8.803	51.411
TRT2	29.813	16.079	13.734	128.414	31.601	540.813	12.149	21.772	2.291	51.087	300.206	1.506	2.71	93.692	197.252	8.758	49.89
TRT2	28.026	16.49	11.536	214.065	60.479	170.457	10.715	21.771	2.424	69.654	358.968	1.026	2.866	94.412	167.426	8.825	45.545
TRT2	28.086	14.855	13.231	152.521	32.274	392.613	9.667	20.294	1.705	91.841	235.847	0.896	2.316	97.092	172.943	8.669	46.311
TRT2	28.576	15.588	12.988	139.366	46.899	661.787	10.081	22.252	2.417	73.571	322.571	1.165	2.858	81.484	189.847	8.825	43.919
TRT2	26.31	16.498	9.812	185.354	37.384	399.825	12.757	24.099	2.035	77.351	538.137	0.884	3.086	93.931	176.329	9.145	44.29

Table S3 Relative abundance of microbial communities at the phylum level.

	Firmicutes	Acidobacteriota	Proteobacteria	Chloroflexi	Actinobacteriota	Bacteroidota	Gemmatimonadota	Crenarchaeota	Cyanobacteria	Verrucomicrobiota	Others
CON	0.0020078	0.18329163	0.34027818	0.11150556	0.18721993	0.04681953	0.02904033	0.00011639	0.00916604	0.02610138	0.06445324
CON	0.00023279	0.12701507	0.40083222	0.0435605	0.15742303	0.15361113	0.04196008	0.0000291	0.00072746	0.02918582	0.0454228
CON	0.00096025	0.29171274	0.26581505	0.16335913	0.09454112	0.00180411	0.05092242	0.04757609	0.00096025	0.01420008	0.06814875
CON	0.94689519	0.0000291	0.00029099	0	0.04839085	0.00014549	0	0	0.00069836	0	0.00355002
CON	0.00250247	0.22554269	0.20991678	0.25627073	0.17892685	0.00171681	0.01353082	0.01254147	0.00253157	0.02496654	0.07155328
CON	0.00177501	0.12145725	0.27806553	0.22624105	0.20738521	0.01568411	0.01923413	0.01388	0.02208578	0.01725543	0.07693651
CON	0.00116394	0.13495897	0.3339638	0.13923645	0.21975208	0.00852587	0.0375662	0.00774021	0.01044637	0.0276436	0.0790025
CON	0.00628528	0.20392248	0.22452424	0.13964383	0.11185474	0.00672176	0.09122388	0.02982599	0.00087296	0.02479195	0.16033289
CON	0.01300704	0.16239888	0.29409882	0.11796543	0.12541465	0.03110633	0.09378455	0.01175581	0.00165862	0.00611069	0.14269918
CON	0.00945702	0.18343712	0.19117733	0.29264389	0.13324216	0.00459757	0.06864343	0.0453646	0.00378281	0.00974801	0.05790607
TRT1	0.0099517	0.18599779	0.19542571	0.35203399	0.09966246	0.01297794	0.03000058	0.03934121	0.00128034	0.02412268	0.04920561
TRT1	0.00922423	0.19603678	0.29724146	0.08944887	0.12893558	0.00727463	0.09067101	0.05394867	0.00072746	0.0197579	0.1067334
TRT1	0.00779841	0.18893674	0.24273992	0.16333004	0.26302159	0.00547052	0.03075714	0.00151312	0.0178083	0.00442298	0.07420125
TRT1	0.00130943	0.24466042	0.2984636	0.14921725	0.09526858	0.00724553	0.07091311	0.05380318	0.00232788	0.00637258	0.07041844
TRT1	0.00058197	0.14328115	0.32744573	0.16856777	0.14866438	0.00384101	0.063202	0.07332829	0.00110574	0.00483036	0.0651516
TRT1	0.00160042	0.08226154	0.30771693	0.09724728	0.14985742	0.02982599	0.12203922	0.00020369	0.04981668	0.06532619	0.09410464
TRT1	0.00020369	0.1778793	0.28947215	0.25822033	0.12753885	0.00110574	0.06032125	0.02583949	0	0.00471396	0.05470523
TRT1	0.00157132	0.14331025	0.26034453	0.23846243	0.12579293	0.01757551	0.03771169	0.01850666	0.07280452	0.02822557	0.05569458
TRT1	0.00078566	0.15058488	0.20790898	0.27256591	0.21023686	0.00235698	0.0275854	0.07661642	0.00040738	0.01492754	0.03602398
TRT1	0.00151312	0.25903509	0.24573707	0.15687016	0.1738928	0.00645987	0.03838096	0.01149392	0.01536402	0.01318163	0.07807135
TRT2	0.00177501	0.25368096	0.2943898	0.08662632	0.18943141	0.0079148	0.03398708	0.00061107	0.00532503	0.04233836	0.08392015
TRT2	0.00058197	0.22752139	0.28341966	0.06643194	0.16327184	0.01821568	0.05822615	0.01903044	0.01466566	0.04841995	0.10021533
TRT2	0.00087296	0.17284525	0.28787173	0.18206949	0.10917768	0.00014549	0.05246465	0.07955537	0.00037828	0.00806029	0.10655881
TRT2	0.00046558	0.23197346	0.22062504	0.25330268	0.1146482	0.0020078	0.04076704	0.06928359	0.00122214	0.00459757	0.06110691

TRT2	0.00078566	0.25918059	0.21497992	0.2133504	0.17069196	0.00474306	0.02810918	0.00657627	0.01309434	0.01597509	0.07251353
TRT2	0.00032008	0.21465984	0.25944247	0.1639411	0.14805331	0.00296805	0.06494791	0.05653844	0.00430658	0.02042717	0.06439504
TRT2	0.00046558	0.42059012	0.1679858	0.15326194	0.10722807	0.00174591	0.03177559	0.03878834	0.00378281	0.02095094	0.0534249
TRT2	0.01420008	0.14042949	0.25874411	0.15905255	0.20185649	0.00634348	0.08683001	0.00808939	0.00317174	0.01155212	0.10973055
TRT2	0.98475237	0	0.00218239	0.0000873	0.01041727	0.0000291	0.0000582	0	0.00029099	0	0.00218239
TRT2	0.98897166	0.0000291	0.00322994	0.00011639	0.00459757	0.00034918	0	0	0	0	0.00270616

Table S4 Relative abundance of microbial communities at the family level.

	Lactobacillaceae	JG30KFAS9	Clostridiaceae	Acidothermaceae	Gemmatimonadaceae	Chitinophagaceae	Sphingomonadaceae	Nitrosotaleaceae	Ktedonobacteraceae	SCI84
CON	0	0.03861375	0.0000291	0.03483094	0.02904033	0.0078566	0.05199907	0.0000873	0.03279404	0.01882675
CON	0	0.00558692	0	0.02060176	0.04187278	0.11933306	0.04329861	0	0.00977711	0.04972938
CON	0	0.05674213	0	0.03602398	0.05092242	0.00107665	0.0058488	0.04681953	0.03744981	0.00305535
CON	0.84135483	0	0.00023279	0	0	0	0	0	0	0
CON	0	0.13222371	0.0000582	0.07888611	0.01353082	0.00096025	0.01355991	0.0118431	0.05584007	0.00128034
CON	0	0.09532678	0	0.06308561	0.01923413	0.00413199	0.02307513	0.01283245	0.0710877	0.00311354
CON	0	0.02333702	0.0000582	0.04216377	0.03730431	0.00625618	0.05028226	0.00683815	0.03096083	0.01888494
CON	0	0.03832276	0.00157132	0.00666356	0.08912879	0.00337543	0.01655706	0.00739103	0.00162952	0.04268754
CON	0	0.04524821	0.00119304	0.00619799	0.09227143	0.01745912	0.02741081	0.0020078	0.00069836	0.07059303
CON	0	0.21192458	0.00541233	0.01434557	0.06698481	0.00407379	0.02904033	0.03369609	0.00421929	0.02906943
TRT1	0	0.28100448	0.00453937	0.01126113	0.02930222	0.00692545	0.0158878	0.0336379	0.0078275	0.03486004
TRT1	0	0.03122272	0.00544142	0.00910784	0.08950707	0.00529593	0.03093173	0.04472444	0.00113484	0.05453064
TRT1	0	0.0158005	0.00017459	0.06771227	0.03069895	0.00069836	0.0198452	0.00093115	0.01268696	0.01932142
TRT1	0	0.10111738	0	0.02339522	0.07079672	0.0040156	0.01129023	0.05307571	0.01000989	0.0217075
TRT1	0	0.10853751	0	0.02746901	0.06302741	0.00273526	0.02092184	0.05714951	0.00756562	0.03058255
TRT1	0	0.02359891	0	0.02130012	0.12203922	0.00977711	0.07740208	0	0.04827446	0.01332713
TRT1	0	0.21890822	0.0000582	0.03232846	0.06032125	0.00098935	0.00363732	0.02301694	0.0038992	0.01737182
TRT1	0	0.12774254	0.0000582	0.03291043	0.03771169	0.00413199	0.04760519	0.01847757	0.06774137	0.00488855
TRT1	0	0.22295292	0.0000582	0.13190363	0.0275854	0.00084386	0.0157714	0.07617995	0.03436536	0.00069836
TRT1	0	0.0651516	0.0000582	0.04996217	0.03838096	0.00436478	0.02694524	0.01149392	0.03017517	0.01239597
TRT2	0	0.01999069	0.0000582	0.04126171	0.03398708	0.00491765	0.03218297	0.00055287	0.01812838	0.02426817
TRT2	0.0000582	0.00884595	0.00014549	0.02301694	0.05822615	0.0139091	0.02633417	0.00290985	0.01452017	0.04434616
TRT2	0	0.0968981	0	0.04772159	0.05246465	0.0000291	0.00340453	0.07658732	0.01853576	0.00346272

TRT2	0	0.14281557	0	0.03841006	0.04076704	0.00064017	0.01242507	0.06113601	0.01559681	0.00279346
TRT2	0	0.05109701	0	0.03518012	0.02810918	0.00264797	0.01961241	0.00360822	0.06651923	0.01035908
TRT2	0	0.08979806	0	0.03806087	0.06494791	0.00145493	0.02447186	0.05403597	0.01932142	0.00686725
TRT2	0	0.06276552	0	0.03288134	0.03177559	0.00107665	0.01117383	0.03768259	0.04446255	0.00372461
TRT2	0	0.05896338	0.01530247	0.02941521	0.04401304	0.00309715	0.01791480	0.02642121	0.02204677	0.01597502
TRT2	0	0.05845894	0.00381191	0.00820578	0.08584066	0.00320084	0.03151371	0.00128034	0.00133853	0.04795437
TRT2	0.80317756	0	0.13370773	0	0	0	0.00011639	0	0	0

Table S5. Effects of *Portulaca oleracea* supplementation in health care sand on metabolites in tumbler pigeons

	Agmatine	Pyropheophorbide-a	Indolelactic acid	N-Acetylmuramate	4-Hydroxyaniline	Thiamine	2'-Deoxycytidine	DL-Mannitol	N-acetylcitrulline
CON	12695723.08	35004572.3	4824879226	50512780.59	40555921.98	16948036.04	13270292.1	4259634061	18932524.66
CON	13740536.19	30959637	8121869519	37419880.54	39212990.34	14191188.89	7573625.603	2545150527	23185939.58
CON	11248510.25	38139705.45	6909706973	15949727.77	40876893.51	15084803.93	7091403.621	2836401777	15623713.77
CON	21212452.81	16089112.05	3719295308	57871270.14	37828152.52	39646997.63	12504076.95	6495369016	20529108.18
CON	15815331.43	24336613.12	4841980084	44650962.68	50110058.18	38485387.72	16661700.67	8633964613	27387581.46
CON	9155482.773	18878703.48	5625366965	18289909.22	47667778.06	16305963.24	8772078.502	4453914527	20805565.09
CON	13304328.99	12303208.06	4143002112	38960373.43	50005721.71	24282396.12	10487193.57	5184745859	33650655.59
CON	45464747.81	22142700.32	4596746940	29845968.29	38802538.68	45062648.57	5730529.054	3481935696	5880135.181
CON	24134149.75	38038570.41	3628883792	46729472.92	31184490.25	50390502.8	12359905.45	3734796443	6716905.991
CON	80812742.95	43961359.49	3180139712	22367223.07	29511065.44	65725282.3	4093769.69	1004563535	3324224.449
TRT1	28078141.94	49574412.35	2928778733	29772923.05	31168244.06	81010076.83	8576479.615	4384536969	4889178.963
TRT1	31212435.28	27277938.6	3319471688	45650836.07	27645591.08	32256303.89	9467803.921	6292151348	27357891.26
TRT1	12583581.3	52275750.48	3492607834	18415835.05	37618965.97	8681090.141	6757609.716	2287900988	10139372
TRT1	94231370.85	15998877.31	3971426001	33340044.8	29543421.21	58536550.54	8852966.113	1815954529	5077402.298
TRT1	43280033.61	27759314.15	2599913984	30501615.98	35364793.28	20239357.35	9215337.514	4017802363	10877797.71
TRT1	11651233.95	86137680.61	1804409226	36844694.57	43712423.21	11937211.81	6065026.992	1426509431	5994388.632
TRT1	49679354.32	33226187.45	3281872704	14541850.19	29995258.67	85740677.97	12039289.31	2071828242	1229370.249
TRT1	89984606.87	29742567.81	3072734466	22298029.12	20175029.12	70000754.76	11375118.9	1225941944	1051572.084
TRT1	20507877.13	28093732.88	4829193288	68434178.28	36417618.97	8875099.626	8575316.981	3135279613	19627915.74
TRT1	21003426.18	30320927.04	5431289301	39660567.16	29577406.85	32411699.41	6613039.222	3201835820	10212386.02
TRT2	17694135.74	25349692.68	2356779731	48266491.92	24282189.67	36989736.57	17608789.41	7722464680	66087644.23
TRT2	8272551.808	9392010.848	2770378638	55278433.4	26891547.72	67705300.59	23333647.49	12131664442	105275104.9
TRT2	12685556.8	10584340.39	2817707012	96579735.47	25346019.07	35369771.65	11602597.54	11746436656	53485250.09
TRT2	22988471.66	27940883.77	2442453124	30720447.11	23261079.61	82900974.25	10053212.4	3749402503	7107463.676

TRT2	17360649.32	13905342.03	2727728177	110078430.3	24912699.1	44939852.05	31661767.66	13684564769	57294837.4
TRT2	26766249.17	10681872.48	2612366396	27837188.99	28523541.81	85235707.34	17100602.93	8422336173	4511071.233
TRT2	8529333.748	3719744.443	4259899869	65906198.03	24314231.7	69300611.21	16561771.5	15394358057	45455284.87
TRT2	10055336.96	13176058.96	3231996226	87052672.87	32207084.58	58683105.23	11548843.6	12299899162	16893317.69
TRT2	12954506.58	30169068.67	3412693413	136702990.6	21218009.13	43061527.66	15859358.73	8672412178	11008154.27
TRT2	14607973.17	10702110.23	4042356673	54697069.56	27600914.76	59508320.73	11214088.37	9624044108	29105898.04
